# High-throughput sequencing analysis of a “hit and run” cell and animal model of KSHV tumorigenesis

**DOI:** 10.1371/journal.ppat.1008589

**Published:** 2020-06-30

**Authors:** Julian Naipauer, Daria Salyakina, Guy Journo, Santas Rosario, Sion Williams, Martin Abba, Meir Shamay, Enrique A. Mesri

**Affiliations:** 1 Tumor Biology Program, Sylvester Comprehensive Cancer Center and Miami Center for AIDS Research, Department of Microbiology and Immunology, University of Miami Miller School of Medicine, Miami, Florida, United States of America; 2 UM-CFAR/ Sylvester CCC Argentina Consortium for Research and Training in Virally induced AIDS-Malignancies University of Miami Miller School of Medicine, Miami, Florida, United States of America; 3 Daniella Lee Casper Laboratory in Viral Oncology, Azrieli Faculty of Medicine, Bar-Ilan University, Safed, Israel; 4 Neurology Basic Science Division, Sylvester Comprehensive Cancer Center; University of Miami Miller School of Medicine, Miami, Florida, United States of America; 5 Centro de Investigaciones Inmunológicas Básicas y Aplicadas, Facultad de Ciencias Médicas, Universidad Nacional de La Plata, La Plata, Argentina; University of North Carolina at Chapel Hill, UNITED STATES

## Abstract

Kaposi's sarcoma (KS), is an AIDS-associated neoplasm caused by the KS herpesvirus (KSHV/ HHV-8). KSHV-induced sarcomagenesis is the consequence of oncogenic viral gene expression as well as host genetic and epigenetic alterations. Although KSHV is found in all KS-lesions, the percentage of KSHV-infected (LANA+) spindle-cells of the lesion is variable, suggesting the existence of KS-spindle cells that have lost KSHV and proliferate autonomously or via paracrine mechanisms. A mouse model of KSHVBac36-driven tumorigenesis allowed us to induce KSHV-episome loss before and after tumor development. Although infected cells that lose the KSHV-episome prior to tumor formation lose their tumorigenicity, explanted tumor cells that lost the KSHV-episome remained tumorigenic. This pointed to the existence of virally-induced irreversible oncogenic alterations occurring during KSHV tumorigenesis supporting the possibility of hit and run viral-sarcomagenesis. RNA-sequencing and CpG-methylation analysis were performed on KSHV-positive and KSHV-negative tumors that developed following KSHV-episome loss from explanted tumor cells. When KSHV-positive cells form KSHV-driven tumors, along with viral-gene upregulation there is a tendency for hypo-methylation in genes from oncogenic and differentiation pathways. In contrast, KSHV-negative tumors formed after KSHV-episome loss, show a tendency towards gene hyper-methylation when compared to KSHV-positive tumors. Regarding occurrence of host-mutations, we found the same set of innate-immunity related mutations undetected in KSHV-infected cells but present in all KSHV-positive tumors occurring en exactly the same position, indicating that pre-existing host mutations that provide an *in vivo* growth advantage are clonally-selected and contribute to KSHV-tumorigenesis. In addition, KSHV-negative tumors display *de novo* mutations related to cell proliferation that, together with the PDGFRAD842V and other proposed mechanism, could be responsible for driving tumorigenesis in the absence of KSHV-episomes. KSHV-induced irreversible genetic and epigenetic oncogenic alterations support the possibility of “hit and run” KSHV-sarcomagenesis and point to the existence of selectable KSHV-induced host mutations that may impact AIDS-KS treatment.

## Author summary

KSHV-infected KS lesions are composed of latently-infected cells, as well as cells expressing lytic genes that have been implicated in the development of the KS angioproliferative phenotype. The existence of KS lesions with varying levels of KSHV-infected cells suggests also the existence of virus-independent “hit and run” mechanisms of sarcomagenesis, whereby viral infection irreversibly induce genetic or epigenetic oncogenic alterations in host cells. We used the unique mECK36 animal model of multistep KSHV sarcomagenesis to dissect transcriptional, genetic and epigenetic mechanisms of KSHV dependent tumorigenesis and during tumorigenesis following KSHV-episome loss (“hit and run”) sarcomagenesis in an unbiased high-throughput fashion. These analyses revealed that KSHV in vivo tumorigenesis: A) Occurs predominantly with CpG hypo-methylation of oncogenic and differentiation pathways. B) Selects for pre-existing host mutations that allow the KSHV oncovirus to express oncogenic lytic genes by creating permissive environment for viral-induced innate immunity and inflammation, which provides a selective advantage *in vivo* conducive to tumorigenesis. Our results highlight the mutagenic potential of KSHV pointing to the existence in KS lesions, of KSHV-induced oncogenic host mutations that could be selected upon treatment and impact AIDS-KS therapies.

## Introduction

Human viral oncogenesis is the consequence of the transforming activity of virally encoded oncogenes in combination with host oncogenic alterations [1]. Kaposi's sarcoma (KS), caused by the Kaposi's sarcoma-associated herpes virus (KSHV), is a major cancer associated with AIDS and is consequently a major global health challenge [2–4]. The KS tumors are characterized by intense angiogenesis and the proliferation of spindle cells that can affect the skin, mucosa and viscera, causing significant morbidity [2–4]. Although KS can be treated with anti-retroviral therapy and chemotherapy, it is estimated that more than a half of AIDS associated KS patients will not be cured [4, 5]. Understanding the interplay between viral and cellular genes leading to KS carcinogenesis is paramount to developing rationally designed therapies for KS.

KSHV-infected KS lesions are composed of a majority of latently infected cells, as well as cells expressing lytic genes that have been implicated in the development of the KS angioproliferative phenotype via paracrine and autocrine mechanisms [2, 3, 6–9]. Like other human oncogenic viruses, KSHV infection alone is generally not sufficient to cause KSHV-associated cancers, as suggested by the very low incidence of KS in the general KSHV-seropositive population [5]. Thus, a critical question emerges: how the interplay between KSHV and host gene expression leads to cell transformation and establishment of the KS angio-proliferative lesion.

DNA methylation at CpG dinucleotides is an epigenetic mark that has been studied extensively in the context of cancer. Methylation of the cytosine residue in the CpG dinucleotide is carried out by the DNA methyltransferases DNMT1, DNMT3a, and DNMT3b [10]. Many promoters contain CpG islands, and these islands are protected from methylation in normal tissues [11]. In cancer cells, some of these CpG islands become aberrantly hyper-methylated, and this is usually correlated with transcription repression [12]. On the other hand, global hypo-methylation has been described in cancer cells as well [13]. DNA methylation is regulated by KSHV on several levels [14]. The latency-associated nuclear antigen (LANA/ORF73) encoded by KSHV leads to CpG methylation by interacting with the cellular *de novo* DNA methyltransferase, DNMT3a, and recruiting DNMT3a to certain cellular promoters that become methylated and repressed [15]. An additional mechanism by which KSHV might modify the human methylome is via the Polycomb complex that creates the histone mark histone H3 trimethylated on Lys27 (H3K27me3) and can direct cellular CpG methylation via its interaction with DNMTs [16, 17]. The pattern of CpG DNA methylation in chronically infected primary effusion lymphoma (PEL) cells and during de-novo in-vitro infection was investigated for the KSHV episomal genome [17–19] and for the host cellular genome [20].

We have developed a cell and animal model based on mouse bone marrow cells of the endothelial cell lineage transfected with a KSHVBac36 recombinant genome (mECK36 tumor model) [21]. A unique feature of the mECK36 cell model is that this tumors show consistent expression of the KS markers and that KSHV tumorigenesis is tightly linked to the presence of the virus, since mECK36 cells that lose the viral episome can survive in culture but are not tumorigenic [21]. Using this mECK36 model we showed that the most prominently activated tyrosine kinase in the tumors was PDGFRA, which was activated by lytic KSHV genes such as vGPCR via a ligand-mediated mechanism, strongly pointing to PDGFRA as a critical oncogenic driver for KSHV sarcomagenesis [22]. More importantly, PDGFRA was prominently expressed and phosphorylated in the vast majority of AIDS-KS tumors [22]. Upon mECK36 tumor formation in mice, explanted cells that are forced to lose the viral episome continue being tumorigenic [23]; in part and as we have recently shown, by the irreversible *in vivo* acquisition of host mutations, as the PDGFRA activating mutation D842V, the most common PDGFRA mutation in GIST, which confers constitutive RTK activity and resistance to Imatinib, supporting the ideas that: 1) PDGFRA is an oncogenic driver in KSHV tumors and 2) Oncogenic mutations may compensate for the loss of KSHV driving tumorigenesis in the absence of the virus [22].

Reports of KS lesions displaying variable percentage of KSHV infected (LANA positive) cells [2, 4, 22] point to the occurrence of KS-spindle cells that have lost the KSHV-episome. This is consistent with studies showing spontaneous KSHV-episome loss in cultures, and the proposed need of continuous re-infection in the KS-lesions and/or paracrine stimulation from infected cells [22, 24, 25]. Yet, since our studies have shown that the KSHV-episome is retained during tumor formation because it provides a growth advantage, the existence of KSHV-negative phospho-PDGFRA positive spindle cells in KS lesions also suggest the possibility of a virus-independent “hit and run” mechanisms of sarcomagenesis. In this “hit and run” scenario, cells would be irreversibly transformed by KSHV and would be able to sustain tumor growth in the absence of the viral episome. This is consistent with some reports that have identified the presence of host oncogenic mutations in KS lesions [26, 27], our own results showing that tumor cells that loose the KSHV virus have a PDGFRAD842V mutation[22], and the isolation of KSHV-negative KS-cell lines such as the KS-Imm[28]. Yet, a mechanistic rationale for a possibility of KSHV induced “hit and run” tumorigenesis have not been yet firmly established. The combination of the mECK36 cells and their derivatives *in vitro* and *in vivo* constitute a unique model to study through the use of Next Generation Sequencing not only the transcriptional events regulated by KSHV *in vitro* and *in vivo*; but more interestingly, to define the viral oncogenic footprint at the DNA level encompassing both changes in CpG methylation as well as mutations in transcribed genes.

In this work, we have integrated genetic mutations analysis, changes in expression signatures and methylation analysis during direct and “hit and run” KSHV oncogenesis *in vitro* and *in vivo*, to dissect genetic and epigenetic signaling pathways in an unbiased manner in the mECK36 mouse model of KSHV tumorigenesis [21]. Pathway analysis of differential expressed genes (DEGs) during KSHV-dependent *in vivo* tumorigenesis showed KSHV lytic gene upregulation and host DNA methylation and Epigenetic regulation as the most regulated pathways during this process. Our methylation analysis data indicates that during the development of tumors the most profound changes are towards hypo-methylation of tissue specific genes and oncogenic signature pathways as well as for KSHV genes. On the other hand, during viral loss and development of KSHV negative tumors the most profound changes are towards hyper-methylation of these and additional oncogenic pathways. Mutational analysis of mECK36 KSHV positive cells and tumors revealed a surprising set of mutations, including mutations in inflammasome related IFN response genes, undetected in KSHV positive cells but present in all KSHV positive tumors in the same location. This result suggests that in the context of *in vivo* tumorigenesis both these mutations and the virus may determine tumor growth. On the other hand, clustering analysis of mutations found in KSHV negative tumors reveal a network, complementary to the PDGFRAD842V mutation, implicated in cell proliferation. Our results have uncovered novel specific aspects of the interplay between host oncogenic alterations and virus-induced transcriptional effects as well as the relationship between epigenetic changes induced by KSHV infection and tumorigenesis. These virally-induced irreversible oncogenic alterations support the possibility of a “hit and run” KSHV sarcomagenesis which is consistent with histological findings and highlight the existence and biological importance of KSHV-induced host mutations that may be selected during AIDS-KS therapies and affects clinical outcomes.

## Results

### Animal Model of Multistep and “Hit-and-Run” KSHV sarcomagenesis

**Fig 1:**
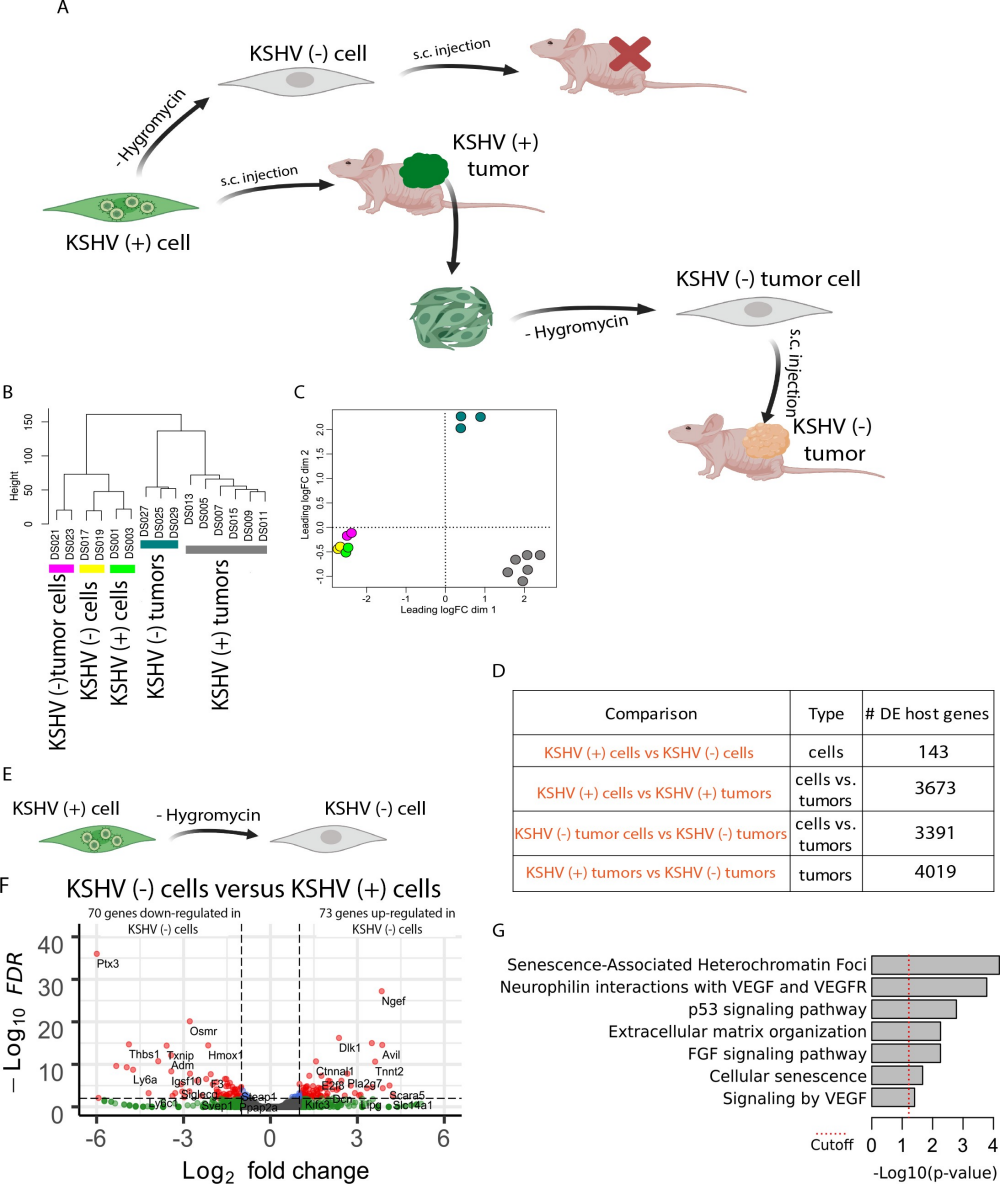
Genome-wide analysis of host transcripts by RNA deep sequencing. **(A)** Schematic representation of the animal Model of Multistep KS Carcinogenesis. **(B)** Unsupervised hierarchical clustering of the host transcriptome. **(C)** Multidimensional scaling plot of the host transcriptome showing the distance of each sample from each other determined by their leading logFC. The leading logFC is a distance metric that represents the average (root mean square) of the largest absolute logFC between each pair of samples. **(D)** Number of differential expressed genes (DEGs) in key biological comparisons that were detected by RNA-sequencing analysis of: two KSHV (+) cells, two KSHV (-) cells, six KSHV (+) tumors, two KSHV (-) tumor cells and three KSHV (-) tumors. **(E)** Schematic representation of the comparison between KSHV (+) cell and KSHV (-) cell. **(F)** Volcano plot showing 144 differentially expressed genes (DEGs) analyzed by RNA-Sequencing between KSHV (-) cells and KSHV (+) cells *in vitro*. 70 genes were down-regulated and 73 gees were up-regulated in KSHV (-) cells. **(G)** Functional enrichment analysis based on the 144 genes differentially expressed among KSHV (-) cells and KSHV (+) cells *in vitro*.

Mouse bone-marrow endothelial-lineage cells (mEC) transfected with the KSHVBac36 (mECK36 cells, abbreviated here as **KSHV (+) cells**) are able to form KSHV-infected tumors in nude mice, which were thoroughly characterized as KS-like tumors [21], thus providing a platform to dissect molecular mechanisms of tumorigenesis by KSHV. Tumors formed by KSHV (+) cells are all episomally infected with KSHVBac36 [21](abbreviated here as **KSHV (+) tumors**). When mECK36 KSHV (+) cells lose the KSHV episome *in vitro* by withdrawal of antibiotic selection (abbreviated as **KSHV (-) cells**), they completely lose tumorigenicity [21]. In contrast to KSHV (-) cells, cells explanted from KSHV (+) mECK36 tumors and grown in the absence of antibiotic lose the KSHV episome (abbreviated as **KSHV (-) tumor cells),** are tumorigenic and are able to form KSHV-negative tumors (abbreviated as **KSHV (-) tumors**) with 100% efficiency and somehow faster than KSHV (+) cells (tumors arising in average by day 30 versus tumors arising in average by day 47, S1 Fig). Since mECK36 cells that lose the episome prior to *in vivo* growth (KSHV (-) cells) completely lose their tumorigenicity, these results suggest that during the process of in vivo-restricted tumorigenesis mECK36 cells became irreversibly transformed by KSHV [21, 23]. This is likely due to host genetic and/or epigenetic alterations accumulated during *in vivo* tumor growth that can compensate for KSHV-induced tumorigenicity after loss of the KSHV episomes. We decided to use this unique animal model of multistep KSHV sarcomagenesis (Fig 1A) to dissect transcriptional, genetic and epigenetic mechanisms of KSHV-dependent and during tumorigenesis following KSHV-episome loss (a.k.a “hit and run”) in an unbiased high-throughput fashion.

### RNA sequencing analysis of mECK36 model of KSHV tumorigenesis

To identify changes at the transcriptional level in our murine model of KSHV-infected cells and tumors [21, 23], high throughput RNA sequencing was performed to identify differences in the gene expression profile, allowing us to perform key biological comparisons (Fig 1D). We have performed Illumina, stranded, RNA sequencing analysis of all KSHV stages of this cell and animal model including **KSHV (+) cells**, **KSHV (-) cells**, **KSHV (-) tumor cells**, **KSHV (+) tumors** and **KSHV (-) tumors**. Expression of 16,016 cellular genes in all replicates were identified. KSHV (-) tumor cells and KSHV (-) tumors showed less than 1 KSHV read per million reads (S2 Fig), validating them as negative for KSHV gene expression. Unsupervised clustering (Fig 1B) and Multidimensional scaling plot (Fig 1C) shows how KSHV status and tissue type cluster with each other. Interestingly, cells *in vitro* cluster together whether they are KSHV (+), KSHV (-) or KSHV (-) tumor cells. On the other hand, KSHV (+) and KSHV (-) tumors *in vivo* are separate from each other and from the cells *in vitro* (Fig 1C), further indicating that processes occurring *in vivo* are predominantly distinct from *in vitro* and are distinctly affected by the uniqueness of KSHV-induced tumorigenesis (Fig 1D).

To understand the effect of losing the virus *in vitro* and the concomitant lack in tumor formation of KSHV (-) cells, we compared host gene expression profiles of KSHV (+) cells with KSHV (-) cells (Fig 1E). As expected by the minimal phenotypic *in vitro* differences already described in our previous work [21], the comparison between KSHV (+) and KSHV (-) cells showed only 143 differentially expressed genes (DEG) (Fig 1D, Fig 1F and S1 Table, Tab-A). Pathway analysis of these DEGs showed changes on Cellular senescence, VEGF signaling [21], FGF signaling and p53 signaling (Fig 1G and S1 Table, Tab-FEA1). The small number of host DEG between tumorigenic KSHV (+) cells and non-tumorigenic KSHV (-) cells *in vitro* highlights the importance of the *in vivo* KSHV lytic switch during the process of tumor formation that was formerly characterized as “in vivo-restricted oncogenesis” [21].

**Fig 2:**
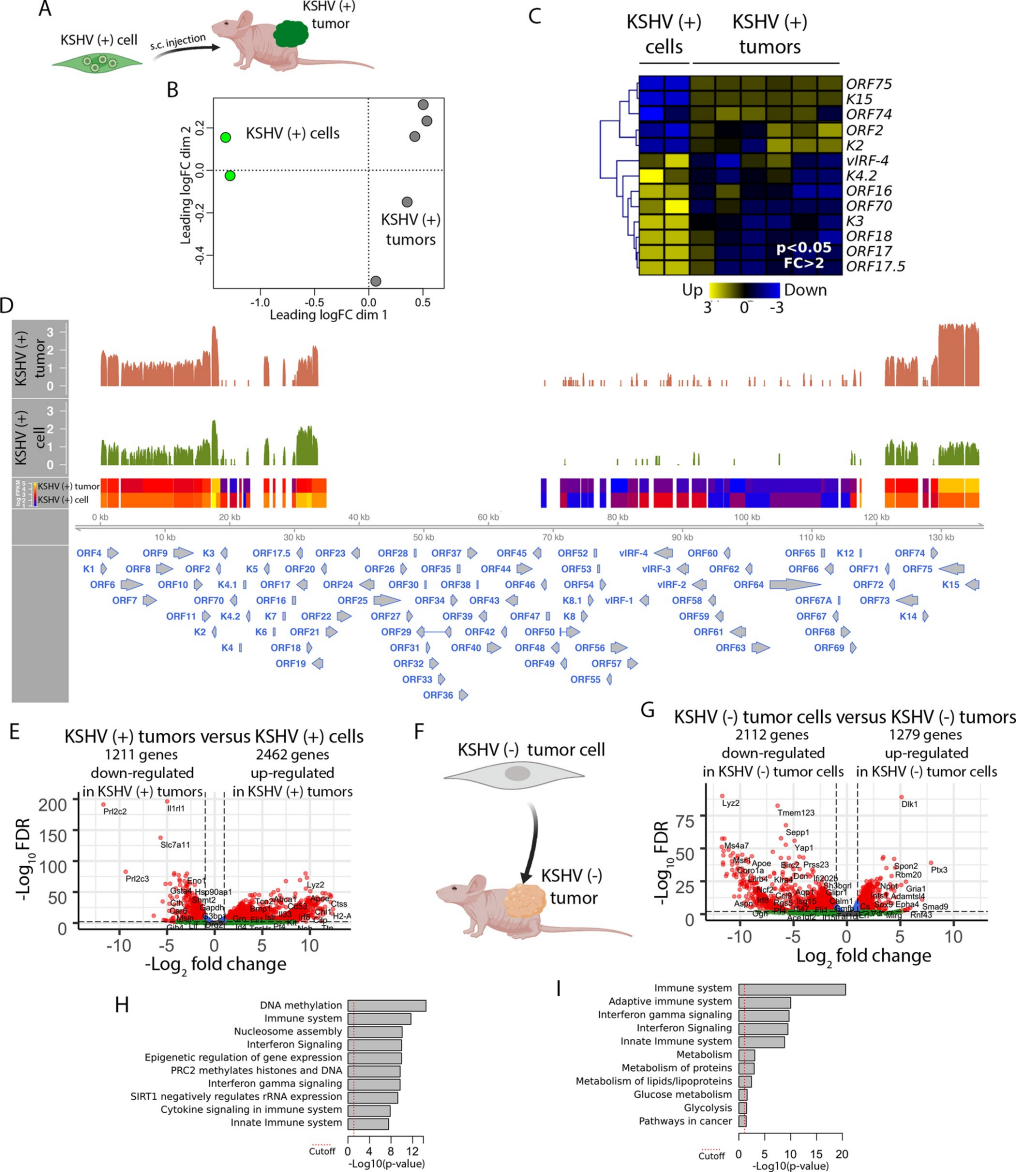
Transcriptional effects mediated by KSHV and by environmental cues *in vivo*. **(A)** Scheme of the comparison between KSHV (+) cell and KSHV (+) tumor. **(B)** Multidimensional scaling plot for KSHV gene expression of KSHV (+) cells and KSHV (+) tumors. **(C)** Heat map for fold change expression of KSHV-encoded genes based on analysis of RNA sequencing data between KSHV (+) cells and KSHV (+) tumors. Only KSHV genes with statistical significance (p<0.05) are shown. **(D)** Histogram showing transcript coverage for KSHV-encoded genes, comparison of the transcription profiles of KSHV (+) cells and KSHV (+) tumors. Transcriptional levels of viral genes were quantified in reads per kilo base of coding region per million total read numbers (RPKM) in the sample. The y-axis represents the number of reads aligned to each nucleotide position and x-axis represents the KSHV genome position. **(E)** Volcano plot showing 3674 differentially expressed genes (DEGs) analyzed by RNA-Sequencing between KSHV (+) tumors and KSHV (+) cells. 1211 genes were down-regulated and 2462 genes were up-regulated in KSHV (+) tumors. **(F)** Scheme of the comparison between KSHV (-) cell and KSHV (-) tumor. **(G)** Volcano plot showing 3392 differentially expressed genes (DEGs) analyzed by RNA-Sequencing between KSHV (-) tumor cells and KSHV (-) tumors. 2112 genes were down-regulated and 1279 genes were up-regulated in KSHV (-) tumor cells. **(H)** Functional enrichment analysis based on the 3674 genes differentially expressed among KSHV (+) cells and KSHV (+) tumors. **(I)** Functional enrichment analysis based on the 3392 genes differentially expressed among KSHV (-) tumor cells and KSHV (-) tumors.

To deepen our understanding of the mechanism of KSHV mediated oncogenesis *in vivo*, we analyzed the KSHV gene expression profiles of KSHV (+) cells and KSHV (+) tumors (Fig 2A). Multidimensional scaling plot and heat map analysis in Fig 2B and 2C shows that these two stages (*in vitro* versus *in vivo*) bear two different KSHV gene expression profiles. KSHV (+) tumors showed an increased expression of lytic KSHV genes (ORF75, ORF-K15, ORF74, ORF-K2), including several well-characterized viral oncogenes such as vGPCR (ORF74), vIL6 (ORF-K2) and K15 (Fig 2C and S1 Table, Tab-B). To better understand and visualize these differences we performed a histogram showing transcript coverage for KSHV encoded genes in KSHV (+) tumors and KSHV (+) cells (Fig 2D). This result corroborates the up-regulation of KSHV lytic genes found *in vivo*, previously described for this model of KSHV tumorigenesis using real-time qRT-PCR array [21, 22] throughout the process of mECK36 tumorigenesis. The lack of RNA-sequencing signal in the region 35-69kb (Fig 2D) suggested a deletion of this portion of the KSHV episome which was later confirmed by qPCR. These type of deletions can occur in Bac36; yet, this did not impacted the capacity of the KSHVBac36 to form tumors [21]. Moreover, we performed KSHV-LANA IFA, in KSHV (+) tumors, and we found the typical speckle nuclear staining pattern indicative of KSHV episomal infection (S3 Fig).

To study and compare transcriptional effects in the host genes induced by *in vivo* environmental cues both in KSHV-dependent tumorigenesis and during tumorigenesis following KSHV-episome loss settings, we compared gene expression profiles of KSHV (+) cells versus KSHV (+) tumors (Fig 2E) with that of KSHV (-) tumor cells versus KSHV (-) tumors (Fig 2F and 2G). Both comparisons showed around 3000 host DEGs (Fig 1D and S1 Table, Tab-B and Tab-C respectively), further indicating the impact of *in vivo* growth conditions on host gene expression. Pathway analysis of these DEGs showed DNA methylation and Epigenetic regulation together with Immune System related pathways as the most differentially regulated when KSHV (+) cells form KSHV (+) tumors (Fig 2H and S1 Table, Tab-FEA2). Interestingly, the transition *in vitro* to *in vivo* but in the absence of KSHV, when KSHV (-) tumor cells form KSHV (-) tumors did not show DNA methylation and Epigenetic related regulation pathways as differentially regulated. Instead they showed Immune and Metabolic related pathways as the most differentially regulated pathways (Fig 2I and S1 Table, Tab-FEA3), further indicating the importance of DNA methylation and Epigenetic regulation during KSHV-dependent transformation and tumorigenic growth.

**Fig 3:**
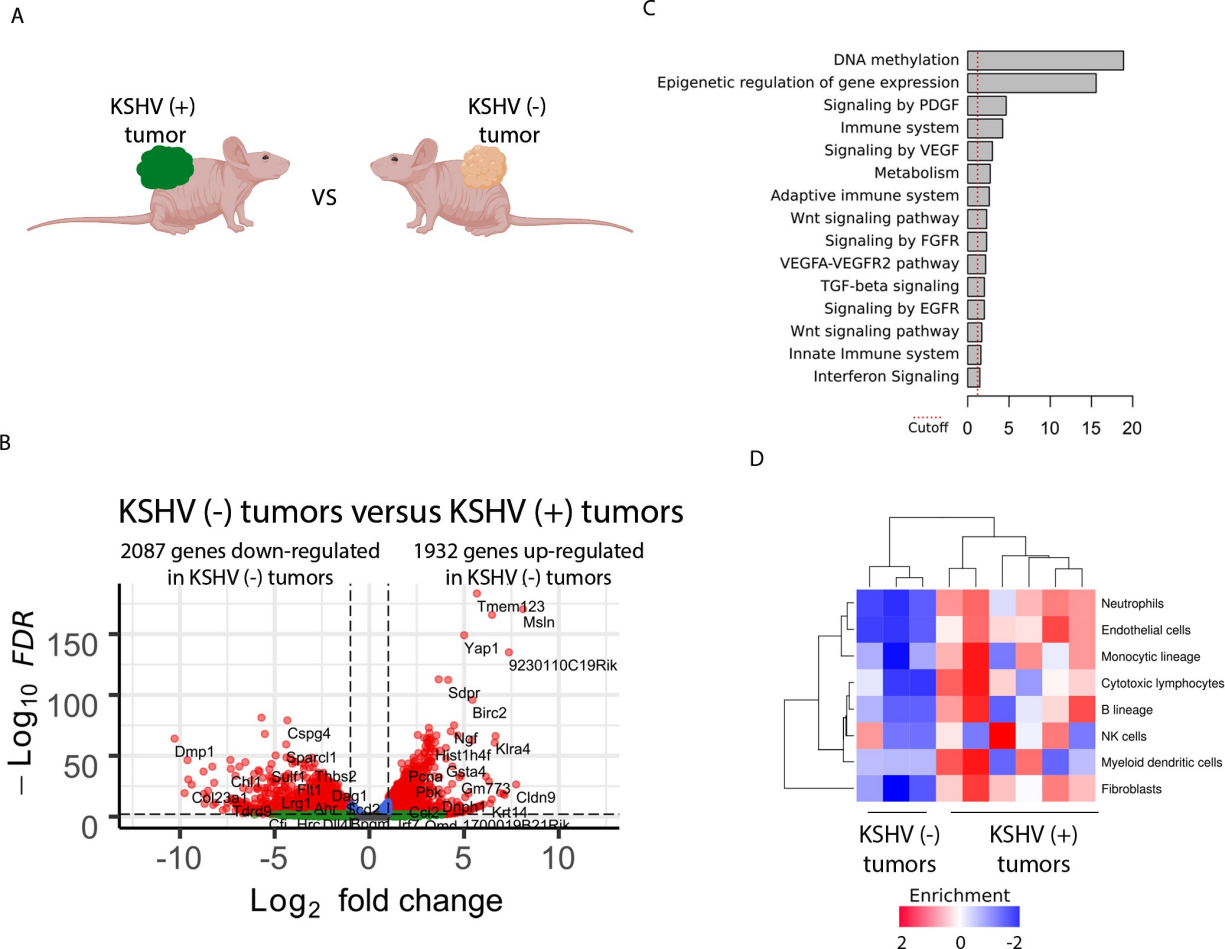
Transcriptional effects of KSHV infection in KSHV-positive and KSHV-negative tumors. **(A)** Scheme of the comparison between KSHV (+) tumors and KSHV (-) tumors. **(B)** Volcano plot showing 4020 differentially expressed genes (DEGs) analyzed by RNA-Sequencing between KSHV (-) tumors and KSHV (+) tumors. 2087 genes were down-regulated and 1932 genes were up-regulated in KSHV (-) tumors. **(C)** Functional enrichment analysis based on the 4020 genes differentially expressed among KSHV (+) tumors and KSHV (-) tumors. **(D)** CIBERSORT *in silico* method to determine absolute immune cell fractions within the tumor microenvironment of KSHV-positive and KSHV-negative tumors.

To study the impact of KSHV infection within KSHV (+) tumors we compared the transcriptome of KSHV (+) tumors with KSHV (-) tumors (Fig 3A), which are both driven by PDGFRA signaling [22]. In KSHV infected tumors PDGFRA is activated by KSHV-induced ligands (PDGFA and B), while in KSHV (-) tumors PDGFRA bears a heterozygous constitutively activated mutated form (D842V)[22]. Fig 3B shows that gene expression profiles differences in KSHV (+) compared to KSHV (-) tumors ascends to 4019 DEG (Fig 1D and S1 Table, Tab-D), illustrating the impact of *in vivo* KSHV gene expression on host gene expression. Remarkably, pathway analysis showed DNA methylation and Epigenetic regulation of gene expression as the most differentially-regulated pathway between KSHV-positive and KSHV-negative tumors (Fig 3C and S1 Table, Tab-FEA4). We used the CIBERSORT *in silico* method [29] to determine absolute immune cell fractions within the tumor microenvironment of KSHV-positive and KSHV-negative tumors. We found the occurrence of Immune cells infiltration (B-cells, neutrophils, NK cells) in KSHV (+) tumors (Fig 3D) which reflects the contribution of the inflammatory infiltrate to KSHV driven tumors by innate immunity mechanisms that can operate even in athymic mice. Although both KSHV (+) and KSHV (-) tumors are highly vascularized, the CIBERSORT showed also a remarkable increase in endothelial cell component which is consistent to the ability of KSHV to upregulate angiogenesis and endothelial specific genes [21] and induce transendothelial differentiation [30, 31].

### Methylation footprint of KSHV infection in the context of KSHV oncogenesis

**Fig 4:**
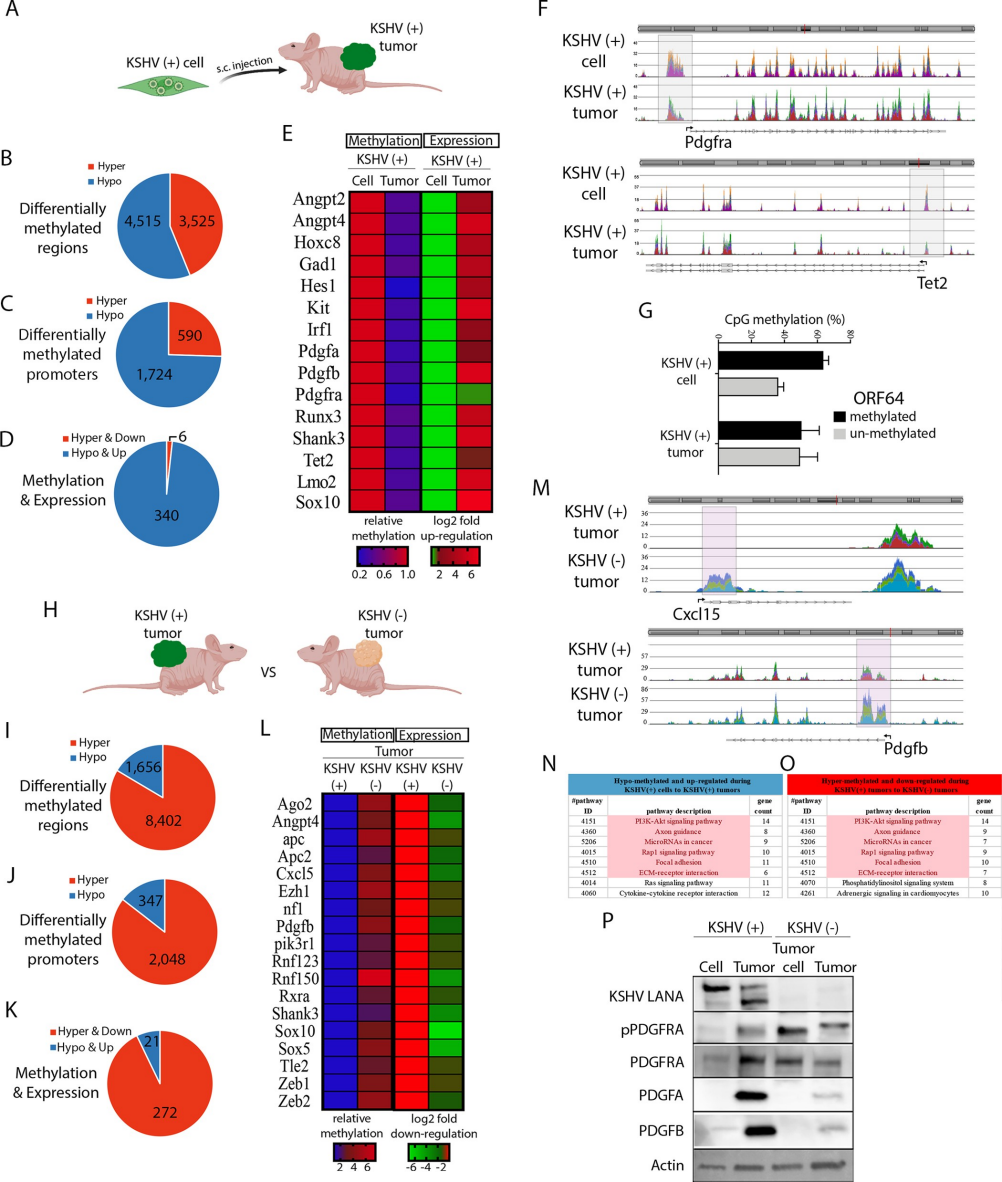
Methylation footprint of KSHV infection in the context of KSHV oncogenesis. **(A)** Scheme of the comparison between KSHV (+) cell and KSHV (+) tumor. **(B)** The number of differentially methylated regions. Red represents hyper-methylation, and blue represents hypo-methylation. **(C)** Differentially methylated promoters (-1500 to +200 relative to TSS). Red represents hyper-methylation, and blue represents hypo-methylation. **(D)** Differentially methylated promoters that were correlated with gene expression. Red represents hyper-methylation, and blue represents hypo-methylation. **(E)** Heat map of selected genes with differentially methylated promoters that were correlated with gene expression in the RNA-sequencing analysis. **(F)** Chromosome view of gene promoters which were differentially hypo-methylated. **(G)** Evaluation of KSHV methylation was performed on MBD2-beads enriched DNA followed by qPCR of the methylated region in ORF64. Percentage of un-methylated (gray) and methylated (black) fractions are presented. **(H)** Scheme of the comparison between KSHV (+) tumors and KSHV (-) tumors. **(I)** The number of differentially methylated regions. Red represents hyper-methylation, and blue represents hypo-methylation. **(J)** Differentially methylated promoters (-1500 to +200 relative to TSS). Red represents hyper-methylation, and blue represents hypo-methylation. **(K)** Differentially methylated promoters that were correlated with gene expression. Red represents hyper-methylation, and blue represents hypo-methylation. **(L)** Heat map of selected genes with differentially methylated promoters that were correlated with gene expression in the RNA-sequencing analysis. **(M)** Chromosome view of gene promoters which were differentially hypo-methylated. (N) Gene ontology analysis on STRING identified pathways that were induced during KSHV-dependent tumorigenesis. Common pathways that were repressed following viral loss are marked in pink. **(O)** Gene ontology analysis on STRING identified pathways that were repressed during tumorigenesis following KSHV-episome loss. Common pathways that were induced following tumorigenesis with the virus are marked in pink. (P) KSHV-LANA, total and phospho-PDGFRA together with PDGFA and PDGFB levels were analyzed by immunoblotting in KSHV (+) cells, KSHV (+) tumors, KSHV (-) tumor cells and KSHV (-) tumors. Actin was used as loading control.

KSHV is a reprogramming virus that encodes viral genes with powerful epigenetic regulating activities that affect the host. In particular, KSHV could affect DNA CpG methylation on the cellular genome, which contribute to cellular transformation and tumorigenicity. A portion of the DNA CpG methylated sites are expected to remain even after KSHV episomal loss leading to irreversible epigenetic regulatory changes affecting host gene expression. We showed in Figs 2 and 3 the importance of DNA methylation related pathways in the process of KSHV-dependent tumorigenesis. DNA methylation analysis of the cells and tumors generated from this multistep model, affords unique biological comparisons and the possibility of studying the footprints of KSHV infection at the level of the CpG methylation landscape. This afforded remarkable and unique observations on the effects of KSHV infection in the host and some of the molecular mechanisms underpinning KSHV oncogenicity. To follow genome wide DNA methylation, DNA from cells grown in culture or during tumorigenic growth in mice, was subjected to enrichment on Methylated DNA binding beads (MBD2-beads) and the eluted DNA served for library preparation and next-generation-sequencing. The sequenced reads were aligned to the mouse (mm10) genome, and enriched peaks were identified, and annotated. During the transition between KSHV (+) cells and their KSHV (+) tumors (Fig 4A), where we showed a KSHV *in vivo* lytic switch and DNA methylation as the most differentially regulated pathway (Fig 2), we identified 4515 differentially hypo-methylated regions and 3525 differentially hyper-methylated regions (Fig 4B, S2 and S3 Tables). In order to correlate differential methylation with gene expression we generated a list of differentially methylated promoter (-1500 to +200) regions. Here, clear preferential for hypo-methylation was observed, with 1724 hypo-methylated and 590 hyper-methylated promoters (Fig 4C, S2 and S3 Tables). Analysis of these hypo-methylated promoter peaks on the GREAT (Genomic Regions Enrichment of Annotations Tool, (http://great.stanford.edu/public/html/index.php) platform identified biological process for pancreatic A cell, astrocyte, dendritic spine, glial cell, enteroendocrine cell, and eye differentiation. In other words, hypo-methylation of many tissue specific promoters, that are expected to be hyper-methylated in the endothelial-lineage cells infected by the virus, and may suggest loss of cell identity/ de-differentiation. These hypo-methylated promoter peaks also identified oncogenic signatures, for genes up-regulated by NF-kB, K-RAS, IL-2, and by knockdown of EED. No terms were identified in biological processes and oncogenic signatures for hyper-methylated promoter peaks (S4 Table). Our data indicates that during the development of tumors and KSHV *in vivo* lytic switch the most profound changes are towards hypo-methylation of tissues specific genes and oncogenic signature pathways. Next, we combined our differential promoter methylation data with gene expression data from Fig 2. This analysis identified 340 hypo-methylated and up-regulated genes, and only 6 hyper-methylated and repressed genes (Fig 4D and S5 Table). Here again, the preference towards hypo-methylation and up-regulation was highlighted. The heat map of several genes that were both hypo-methylated and up-regulated in KSHV (+) tumors is presented (Fig 4E). These include the two Platelet Derived Growth Factors Pdgfa, Pdgfb and their receptor Pdgfra. In a recent study, we have shown that the KSHV-ligand mediated activation of the PDGF signaling pathway is critical for KS development [22]. Here, we also identified hypo-methylation and up-regulation of Angiopoietin 2 (Angpt2) and 4 (Angpt4) that in combination with VEGF facilitate endothelial cell migration and proliferation, to promote angiogenesis. In addition, hypo-methylation and up-regulation of several neuronal specific, Pax6 and Shank3 genes, linking KSHV-associated tumorigenesis with loss of cell identity. Chromosome view of two genes that we detected hypo-methylation in the regulatory region, clearly show that the loss of DNA methylation is specific in the gene promoter around the transcription start site (Fig 4F). We validated our global methylation analysis by performing qPCR with specific primers for several differentially methylated promoter regions (S4A Fig). Methylation analysis of the KSHV genome showed also a trend to more hyper-methylation in KSHV (+) cells than in KSHV (+) tumors (Fig 4G) correlated with an increase in KSHV viral gene expression in these KSHV (+) tumors (Fig 2). Gene ontology analysis on STRING (https://string-db.org) for the list of hypo-methylated and up-regulated genes, identified the following cellular pathways; Ras signaling, cytokine receptors, PI3K-Akt signaling, Rap1 signaling, calcium signaling, focal adhesion, and ECM-receptor interaction (Fig 4N).

Next, we followed the process where KSHV (+) tumor cells have lost the viral episome by growth without antibiotic selection and re-grown as tumors in mice, forming KSHV (-) tumors (Fig 4H). We identified 8402 differentially hyper-methylated regions and 1656 differentially hypo-methylated regions, with a clear preference for hyper-methylation (Fig 4I, S6 and S7 Tables). When the list of differential methylation was limited to promoter (-1500 to +200) regions, again a clear preferential for hyper-methylation was observed with 2048 hyper-methylated and only 347 hypo-methylated promoters (Fig 4J,S6 and S7 Tables). While in the transition between KSHV (+) cells to KSHV (+) tumors hypo-methylation was more significant (Fig 4B–4D), in the comparison between KSHV (+) tumors with KSHV (-) tumors hyper-methylation governs (Fig 4I–4K). Analysis of these hyper-methylated promoter peaks on the GREAT (Genomic Regions Enrichment of Annotations Tool, (http://great.stanford.edu/public/html/index.php) platform identified biological process for actin filament organization, regulation of cell fate, cell-substrate and cell-matrix adhesion, phagocytosis, epithelial cell differentiation, smooth muscle contraction, and Rac protein signal transduction. The pathways involved in BCR (B-cell receptor) signaling, RXR (retinoid x receptor) and RAR (retinoic acid receptor) signaling. These hyper-methylated promoter peaks also identified oncogenic signatures for SELL, MYD88, RAGE, LTK, and PML. No terms were identified in these categories for hypo-methylated promoter peaks (S8 Table). Our data indicates that during KSHV loss and development of KSHV (-) tumors the most profound changes are towards hyper-methylation. When we combined our differential promoter methylation data with gene expression data from Fig 3, we identified, 272 hyper-methylated and down-regulated genes, and only 21 hypo-methylated and up-regulated genes (Fig 4K and S5 Table). Here again, the preference towards hyper-methylation and down-regulation was highlighted. The heat map of several genes that were both hyper-methylated and down-regulated in KSHV (-) tumors are presented (Fig 4L). These include some examples of genes that were hypo-methylated and up-regulated during the transition from KSHV (+) cell to KSHV (+) tumor and are now hyper-methylated and down-regulated, such as Angpt4, Pdgfb, and Shank3. The negative regulators of the WNT signaling, APC and APC2, and Tle2 a transcriptional corepressor that Inhibits the Wnt signaling via interaction with CTNNB1 and TCF. This highlights the need to activate the Wnt signaling by other means, following the loss of the virus. Down-regulation for the positive regulators of the epithelial/endothelial to mesenchymal transition, Zeb1 and Zeb2, that are in agreement with a shift backward from mesenchymal to epithelial/endothelial following viral loss. In addition, regulators of RNA interference, Ago2, and regulators of chromatin organization, polycomb catalytic subunit Ezh1. Chromosome view for examples of two genes with hyper-methylation in the regulatory region, clearly show increase in DNA methylation in the gene promoter (Fig 4M). We validated our global methylation analysis by performing qPCR with specific primers for several differentially methylated promoter regions (S4B Fig). Gene ontology analysis on STRING (https://string-db.org) for these hyper-methylated and down-regulated genes identified genes in the following cellular pathways (KEGG); PI3K-Akt signaling, Rap1 signaling, focal adhesion, ECM-receptor interaction, adrenergic signaling in cardiomyocytes, cholinergic and glutamatergic synapse (Fig 4O). Interestingly, pathways including PI3K-Akt signaling, Rap1 signaling, axon guidance, microRNA in cancer, focal adhesion, and ECM-receptor interaction were upregulated and their gene promoters were hypo-methylated during tumorigenesis with the virus, and these same pathways were down-regulated and gene promoters were hyper-methylated during tumor formation following loss of the virus (Fig 4N and 4O). This indicates that viral proteins/RNAs are necessary for both the hypo-methylation and up-regulation, but also for the maintenance of these pathways’ activation. These marks are eliminated following viral loss. In order to validate our RNA-sequencing, pathway analysis and DNA methylation data we performed Western blot assays to study the PDGFRA activation axis (Fig 4P). We found PDGFRA activation and upregulation of its ligands in the transition *in vitro* to *in vivo*, when KSHV (+) cells form KSHV-dependent tumors as previously shown [22]. This upregulation correlates with our methylation and expression data showing hypo-methylation and upregulation of Pdgfa, Pdfgb and Pdfgra in KSHV (+) tumors (Fig 4E). On the other hand, in the transition *in vitro* to *in vivo* but in absence of KSHV, when tumor cells that lost the KSHV episome form KSHV (-) tumors, we did not found increase in the activation of PDGFRA signaling, with very low level of the PDGF ligands (Fig 4P). This data correlates with the methylation and expression data showing hyper-methylation and downregulation of Pdgfb in KSHV (-) tumors when compared with KSHV (+) tumors (Fig 4L), as well as with previous report showing that in KSHV (-) tumors PDGFRA bears a heterozygous constitutively activated mutated form (D842V) [22].

### Analysis of the mutational landscape

**Fig 5:**
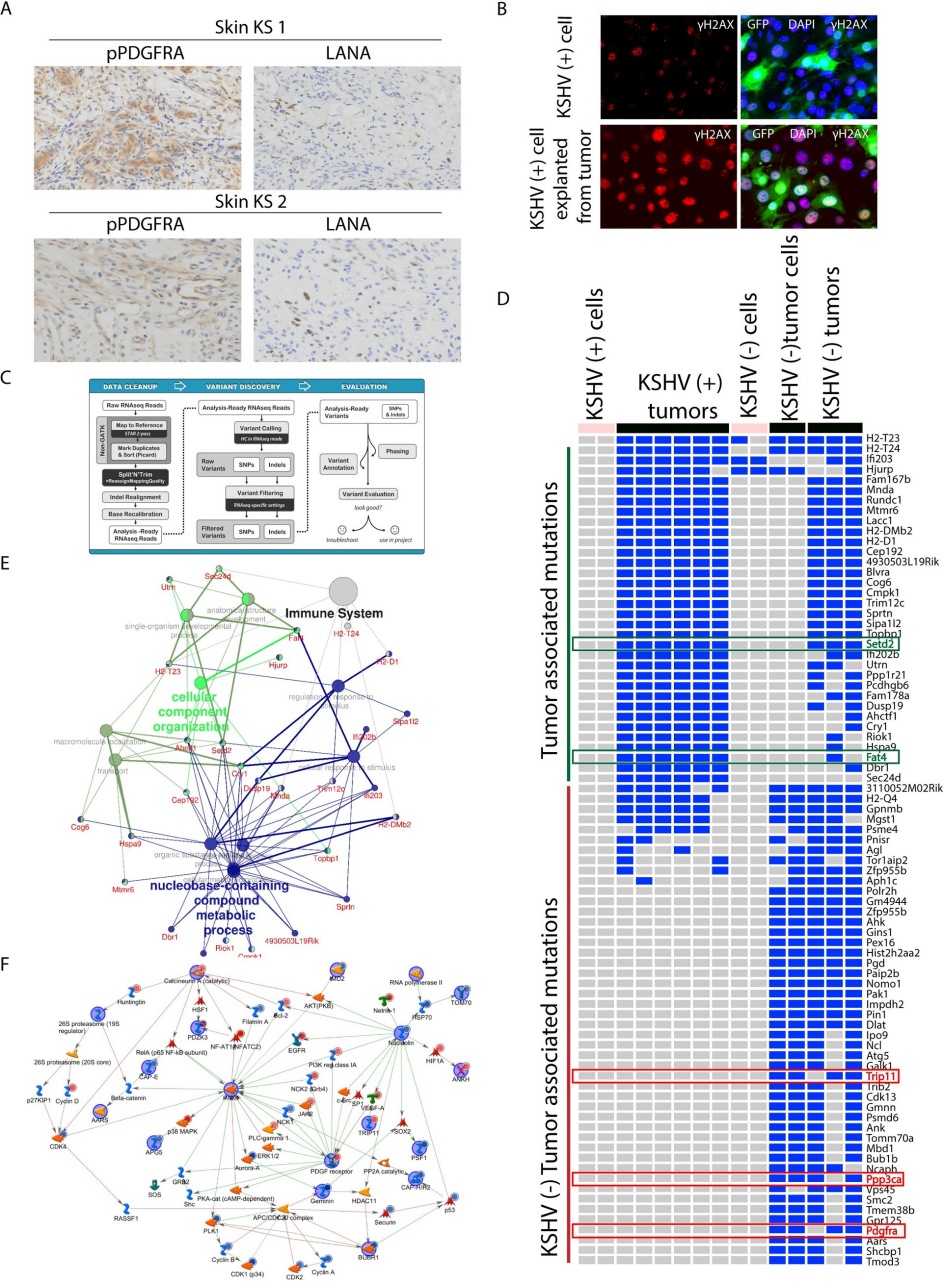
Analysis of the mutational landscape. **(A)** Staining of AIDS-KS biopsies from an ACSR tissue microarray (TMA) showing high phospho-PDGFRA and low LANA staining in two characteristic samples. **(B)** Immunofluorescence analysis of ƴH2AX expression (red) was performed on GFP-positive KSHV (+) cells and GFP-positive KSHV (+) cells explanted from tumor; nuclei were counterstained with DAPI (blue). **(C)** The GATK workflow used to call host mutations. **(D)** Heat map of the most frequently and highly mutated genes. Genes with driver mutations in cancer are highlighted. **(E)** Network analysis of mECK36 KSHV (+) tumors associated mutations (tumor associated mutations). **(F)** Network modeling with genes mutated in KSHV (-) tumor associated mutations.

KSHV-negative tumors belong to the same cell lineages as mECK36 tumors and share a significant overlap with the human KS transcriptome [23]. Therefore, they are the best available control to be used in combination with KSHV-positive tumors, which are a model of KSHV-dependent tumorigenesis [21], to assess KSHV-specific biology [23]. As discussed above; in contrast to KSHV (+) cells that when they lose the KSHV episome lose their tumorigenicity, explanted KSHV (+) tumor cells that lose the episome are strongly tumorigenic. This likely indicates that during *in vivo* tumorigenic growth mECK36 cells acquire irreversible host oncogenic alterations, such as oncogenic mutations, that are able to convey tumorigenicity in the absence of KSHV. We have previously found one such mutation as KSHV (-) tumors and cells had a heterozygous D842V mutation in the tyrosine kinase (TK) domain of the PDGF receptor alpha [22]. In KSHV (+) tumors and cells, PDGFRA was wild-type and activated by its ligands PDGFA/B that were induced via a KSHV-dependent mechanism [22]. We found that the PDGFRAD842V mutation, which was described in GIST as a driver mutation, also was a driver mutation in our KSHV (-) tumors further supporting the idea that PDGFRA is a key oncogenic driver in KSHV tumors, that in its activated mutated form can compensate for KSHV loss [22]. This findings also suggested the possibility that such scenario may occur in KS lesions in which host oncogenic alterations induced through KSHV oncogenic activity may compensate for KSHV loss. In fact, we were able to identify skin AIDS-KS biopsies in which only a small percentage of phospho-PDGFRA positive KS spindle-cells were LANA positive (Fig 5A). The existence of these KS lesions with low levels of KSHV infected cells, together with other reports showing that the proportion of infected cells is variable in KS lesions [2, 4], suggests the possibility of a virus-independent “hit and run” mechanisms of sarcomagenesis whereby the KSHV oncovirus is able to induce irreversible genetic and epigenetic alterations. To assess the possibility that during *in vivo* tumorigenic growth, KSHV driven mutagenic mechanisms such as oxidative stress and DNA damage repair impairment [23, 32, 33] may inflict mutagenic damage in the host genome, we compared the levels of DNA repair foci between mECK36 KSHV (+) cells and KSHV bearing mECK36 cells “just explanted” from tumors. Fig 5B shows that KSHV (+) cells explanted from KSHV (+) tumors have much higher levels of ƴH2AX phosphorylation than their cultured counterparts, which indicates that during the tumor growth KSHV (+) cells increased their foci of DNA repair. This suggests that during tumorigenic *in vivo* growth, there is an increase in DNA damage and; thus, KSHV (+) cells explanted from tumors are likely to have acquired irreversible host tumorigenic mutations that may contribute to overall tumor growth and may allow explanted cells to form tumors in the absence of KSHV.

In order to analyze mutations acquired during *in vivo* tumorigenic growth we used the RNA-sequencing data and the Genome Analysis Toolkit (GATK) workflow to call for host mutations (Fig 5C). Detailed analysis of the mutational landscape of KSHV (+) and KSHV (-) cells and tumors was carried out taking in account the following criteria: 1) Transcripts have to be expressed in all compared samples (seq. depth >5). 2) We selected highly mutated genes: either one homozygous or two heterozygous mutations in various locations of the gene. One remarkable finding was that there were set of genes mutated in all six KSHV (+) tumors, but not in the KSHV (+) cell cultures (Tumor-associated mutations, Fig 5D and S9 Table). Interestingly, 56 genes had recurrent mutations in the exact same position. The only possible explanation for this finding is that these mutations were present already in the polyclonal population of KSHV (+) cells but were not detected by the RNA-sequencing analysis since they were present in a minimal percentage of cells. Yet, the consistent appearance of mutations in exactly the same gene locations in all the tumors once the cells grew *in vivo* indicates that these mutations were enriched as they likely provided an *in vivo* survival advantage. Moreover, many of the mutations that “appear” in all KSHV (+) tumors “disappear” in explanted cells only to “reappear” when these KSHV (-) tumor cells are re-injected *in vivo* to form KSHV (-) tumors, further showing the reversibility of the selection process once the polyclonal population of cells are explanted and how selection operates again when these cells grow back *in vivo* (Fig 5D). Interestingly, network analysis of these mutations showed that many of them corresponded to innate immune genes including TRIM12, several members of the IFI family which are IFN inducible genes (Mnda, IFI203, IFI202) and a series of genes implicated in cytoplasmic DNA sensing and inflammasome/IFN activation (Fig 5E). The analysis of these mutations points to KSHV tumorigenesis as a complex combination of effects from lytic KSHV oncogenesis combined with host mutations in innate immune genes that enable expression of those viral oncogenic genes and repress mechanism of innate immunity against the virus allowing KSHV-dependent tumorigenesis to progress. We found another very interesting set of mutations which appear de novo and manifested only on mECK36 explanted tumor cells that have lost the virus (KSHV (-) tumor cells) and the tumors they form (KSHV (-) tumors) (KSHV (-) Tumor associated mutations, Fig 5D and S9 Table). These were acquired and are the consequence of KSHV oncogenicity during *in vivo* tumors growth. It is unlikely that they are solely a product of selection upon culture in the absence of antibiotic *in vitro* since KSHV (-) cells that were generated in a similar way that KSHV (-) tumor cells are not tumorigenic and freshly explanted KSHV infected tumor cells show increased levels of DDR foci consistent with an *in vivo* mutagenesis scenario (Fig 5B). Clustering analysis of these mutations reveal a network comprising the already described PDGFRA D842V, Pak1 (a Rac1 activated kinase) and Nucleolin mutations implicated in cell proliferation (Fig 5F). It is possible that these mutations would be able to contribute driving tumorigenesis compensating for KSHV loss. In addition to the driver mutation PDGFRAD842V which we previously were able to target in KSHV (-) tumors with the potent PDGFRA inhibitor Sunitinib [22], we analyzed all the mutated genes found in our analysis using the IntOGen-mutations platform to identify genes with driver mutations [34]. Interestingly, in addition to PDGFRA we found four genes previously shown to be driver mutations in different cancers: SETD2, FAT4, TRIP11 and PPP3CA (Fig 5D, highlighted genes).

## Discussion

Viral oncogenesis is the consequence of dysregulation of host signaling proliferation and survival pathways caused by viral oncogenes, in combination with host oncogenic alterations catalyzed by viral infection, that are accumulated through the process of oncogenesis. This “transforming capacity” of viral infection supports the possibility of “hit and run” scenario, whereby episomal viruses that cause cellular transformation through a irreversible genetic alteration—the initial “hit,”—can be lost—they could “run”—since the transformed phenotype would be maintained by the host oncogenic alterations [33]. The hit-and-run hypothesis proposes scenarios in which cumulative host mutations can allow viral genomes to be lost entirely, such that cancers remaining virus-positive represent only a fraction of those to which infection contributes [35]. We have generated a unique multistep KSHV tumorigenesis model in which cells explanted from a KSHV(+) PDGFRA (+) tumor that lose the episome can form KSHV (-) tumors driven by host mutations such as the PDGFRA-D842V [22, 23]. We hypothesize that these KSHV (-) tumors may represent KSHV LANA (-) PDGFRA (+) KS spindle cells that composed many KS tumors, particularly those where KSHV infected cells are a minority of tumors cells [2, 4, 22]. We took advantage of this model and NGS to interrogate the transcriptional, genetic and epigenetic (CpG island methylation) landscape upon KSHV tumor formation and upon KSHV-loss in cells and tumors.

We identified DNA methylation and Epigenetic regulation as the most relevant pathways involved in KSHV-dependent tumorigenesis occurring along with up-regulation of KSHV lytic genes. In contrast, during tumorigenesis following KSHV-episome loss we found Immune and Metabolic regulation as the most relevant pathways involved in this process. We found hypo-methylation of tissue specific genes and oncogenic signature pathways occurring along KSHV-dependent tumorigenesis. Conversely, during viral loss and development of KSHV negative tumors the most profound changes were towards hyper-methylation of these and additional oncogenic pathways. In analyzing the mutational landscape we found a set of mutations in genes regulating viral DNA and innate immunity, which were absent in KSHV-infected cells but present in all KSHV-infected tumors. This indicates that KSHV tumorigenesis not only selects for the presence of the virus but also pre-existing host mutations that only provide a selective advantage *in vivo*. Moreover, we found that KSHV oncogenesis also induced the accumulation of mutations implicated in cell proliferation which appear de novo and manifested only on KSHV (-) tumor cells and KSHV (-) tumors, these mutations would be responsible to be driving tumorigenesis compensating for KSHV loss. This mechanism could work together with other previously described mechanisms showing that virus-induced epigenetic alterations may continue to support proliferation and survival of spindle cells after loss of KSHV [24].

We have previously shown, by real-time qRT-PCR, that KSHV tumorigenesis in the mECK36 mouse KS-like model occurs with concomitant up-regulation of KSHV lytic genes and angiogenic ligands/receptors [21, 22]. After completion of all our metadata analysis, we confirm a deletion between positions 35–69 kb in the KSHV-Bac36 genome. Genome instability has been documented for the Bac36 [36]; yet it does not affected the ability of the Bac36 to be tumorigenic in the mECK36 cells, which reflects also the fact that defective KSHV genomes—that have been found in KS lesions—are oncogenic [37]. Although it could pose a limitation in some of the potential uses of our model, the deletion of the KSHV genome did not impacted the capacity of the KSHVBac36 to form tumors. Furthermore, findings on our recently published work [38] using a novel model of *de novo* KSHV oncogenesis based on infection of PDGFRA-positive mesenchymal stem cell progenitors with rKSHV219 suggest that the deletion occurred in a region of the KSHV genome that is least involved in tumorigenesis. We showed by RNA-sequencing in KS-like mouse tumors that indeed this 35-69kb portion of the KSHV episome is the area of the KSHV genome least expressed in KSHV-induced tumors [38]. Therefore, except for the inability of our model to capture the effects of the KSHV gene expression in this region, the deletion in KSHVBac36 do not affect our capability to study KSHV-dependent tumorigenesis and the effect of KSHV loss. Another indication of the biological value of our mouse system is the fact that allowed us to identify PDGFRA as the main driver of KSHV sarcomagenesis [22]. Importantly, our present RNA-sequencing analysis also showed up-regulation of all the members of the PDGF family members in KSHV (+) tumors together with an increase in KSHV lytic gene expression. Moreover, pathway analysis of differentially expressed genes (DEGs) showed that DNA methylation and Epigenetic regulation are the most relevant pathways involved in KSHV-dependent tumorigenesis (Fig 2H). This indicates that, as expected for a virus with strong epigenetic reprogramming capacities, KSHV-induced tumorigenesis is tightly linked to epigenetic regulation of host gene expression. This is also reinforced by the fact that during tumorigenesis following KSHV-episome loss this process occurred predominantly by Immune and Metabolic pathway regulation (Fig 2I). Epigenetic processes have been heavily implicated in the development of cancer, in which repression or silencing of tumor suppressor genes is remarkably common [14]. Interestingly, KSHV (+) tumors showed up-regulation of Immune cells infiltration and endothelial cell components when compared with KSHV (-) tumors by CIBERSORT analysis, which is consistent with the recruitment of inflammatory cells in the context of viral tumorigenesis and to the ability of KSHV to up-regulate angiogenesis respectively.

Our system allowed following whole genome CpG methylation of the host during tumorigenesis by KSHV. During KSHV-dependent tumorigenesis, we observed clear preference towards hypo-methylation in gene promoters. It is important to mention that the goal of this study was to identify the changes during tumorigenesis and not following infection, therefore the hyper-methylation immediately following infection is already present in the KSHV (+) cells. These results are in agreement with a previous study [20] that tried to address this question indirectly by comparing KSHV-negative BJAB to de-novo infected BJAB (BJAB.219) or to chronically infected PEL cells, and detected very similar hyper-methylation between de-novo infection and PEL, but dramatic difference towards hypo-methylation in PEL. While our study directly evaluates the methylation changes during transformation, both studies support the notion that hypo-methylation is the major epigenetic change during this process. Interestingly, hypo-methylation is also the major process during transformation of naïve B-cells following Epstein-Barr virus (EBV) infection [39].

CpG DNA methylation is an epigenetic mark that can be faithfully maintained between generations [40, 41]. Therefore, CpG methylation changes imposed by viral infection are expected to maintain following viral clearance. Our findings indicate that indeed some methylation changes are maintained following viral clearance, but some are lost, suggesting that the presence of viral encoded proteins or RNAs are necessary to maintain these epigenetic changes. In the case of KSHV (+) tumors these gene promoters are hypo-methylated and the genes are expressed, and in KSHV (-) tumors upon virus loss, these promoters becomes methylated again and repressed.

During the development of tumors following loss of KSHV we observed hyper-methylation of three main groups of genes: 1) genes that were previously hypo-methylated by the virus and viral gene products need to be expressed in order to keep these genes active, therefore viral loss leads to re-repression and hyper-methylation. 2) Oncogenic pathways that are activated by the virus and are essential for tumor growth, upon viral loss the tumor cells need to find alternative ways to activate these pathways. Nice example is the Wnt signaling that is activated by KSHV encoded LANA [42]. During tumor growth without the virus we detected hyper-methylation of three repressors of the Wnt signaling, the tumor suppressors and the antagonists of the Wnt signaling, APC [43] and APC2 [44], and Tle2 a transcriptional corepressor that binds to and Inhibits the transcriptional activation of CTNNB1 and TCF family members that mediate the Wnt signaling [45]. Moreover, we found PDGF signaling members (Pdgfa, Pdgfb and Pdgfra) also hypo-methylated and up-regulated in these transitions as well, correlated with PDGFRA signaling activation (Fig 4P) and further illustrating another KSHV-dependent mechanism to maintain the activation of this oncogenic signaling pathway. 3) Changes due to cell differentiation/de-differentiation. KSHV was found to induce endothelial to mesenchymal (EdMT) transition [30, 46]. In agreement with this transition, we observed hypo-methylation of ZEB1 during tumor development in KSHV infected cells. On the other hand, when tumors develop in the absence of the virus, we observed hyper-methylation of ZEB1 and ZEB2, which indicates a shift backwards from mesenchymal to endothelial (MTEd) transition.

KSHV is a reprogramming virus encoding genes with powerful epigenetic regulatory abilities. Characterization of the CpG methylation footprint of KSHV infection showed a tendency towards hypo-methylation concomitant with KSHV tumorigenesis occurring along the up-regulation of KSHV lytic genes and hyper-methylation in comparing tumors infected versus un-infected with KSHV. The methylation level of the genome is controlled by two opposing activities; DNA methyltransferase, DNMT1, DNMT3a, and DNMT3b, and DNA de-methylases, TET1, TET2 and TET3. Our analysis during tumor development of KSHV (+) tumors detected promoter hypo-methylation and up-regulation of the demethylase *Tet2* gene. This observation can provide a mechanistic explanation for the hypo-methylation that occurs during tumorigenesis. These results showed the importance of DNA methylation regulation in the process of KSHV-dependent oncogenesis.

The existence of spindle-shaped cells within a KS lesion positive for phospho-PDGFRA and negative for KSHV LANA would exemplify a LANA-negative proliferative spindle cells which are not rare in KS, since KS lesions with varying levels of KSHV infected cells, ranging from <10% to >90% of the total cell population in the KS lesions have been reported [2, 4]. This, together with the observation of spontaneous KSHV episome loss in cultures of KS spindle-cells and the isolation of tumorigenic KSHV (-) KS spindle-cells [28], suggests the existence of virus-independent “hit and run” mechanisms of sarcomagenesis whereby the KSHV oncovirus is able to induce irreversible genetic and epigenetic alterations [33, 35]. This is also supported by the fact that KSHV is a reprogramming virus and the occurrence of host oncogenic alterations in KS lesions [26, 27]. We previously found that KS lesions overexpress Rac1 and that oxidative stress plays a major role in KS sarcomagenesis [23, 32]. ROS mediated *in vivo* genetic damage could lead to accumulation of mutations that could contribute to the viral oncogenesis process and may compensate for the loss of virus as found in some KS lesions (Fig 5A). The expression of viral oncoproteins and RNAs may interfere not only with regulators of cell proliferation, but also with DNA repair mechanisms [33]. We found that cells explanted from tumors display much more foci of DNA repair which is consistent with increased “in tumor” DNA damage and DDR (Fig 5B). Genomic instability (GI) is a hallmark of many cancers. This is probably because ongoing mutations associated with GI increase the frequency of oncogenic changes that feed natural selection during tumor progression [47].

Analysis of the mutational landscape revealed a set of genes with recurrent mutations in the exact same position mutated in all six KSHV (+) tumors, but not in the KSHV (+) cell cultures (Fig 5D). This indicates that in the context of in vivo KSHV infection, tumorigenesis not only selects for the presence of the virus [21] but also pre-existing host mutations that—as the KSHV episome—only provide a selective advantage *in vivo*. Interestingly many of these mutations appear to be in genes regulating viral DNA and innate immunity genes. Some of these TRIM and IFI family genes, which are IFN inducible genes, have already shown to be important for KSHV lytic reactivation [48–50]. It is likely that these mutations allow the KSHV oncovirus to express oncogenic lytic genes and that they allow for a permissive environment of inflammatory and viral tumorigenesis, which occurs in the context of DDR that may be impeded by innate immune DNA sensors.

We found that KSHV oncogenesis also induced the accumulation of a set of de novo mutations that were not present in KSHV (+) cells and KSHV (+) tumors. These mutations were first selected upon KSHV loss *in vitro* and would be responsible—together with PDGFRA D842V— to be driving tumorigenesis compensating KSHV loss (Fig 5D). We recently found that the most prominent of these mutations is the one activating the KS oncogenic driver PDGFRA (PDGFRAD842V), that allows the cell to maintain PDGF driven tumorigenesis in the absence of KSHV [22]. Network analysis of the other de novo mutations (KSHV (-) tumor associated mutations) points to the existence of a PDGFRA-Rac1 driven network that is consistent with the Rac1 overexpression in AIDS-KS [32] and mECK36 KSHV (+) and KSHV (-) tumors as well as the proposed role of ROS in AIDS-KS as shown by mECK36 NAC sensitivity [23].

The direct and “hit and run” virally-induced oncogenic mechanisms proposed through our modelling in mECK36 cells provide the basis for an additional source of tumor heterogeneity in KS lesions that may be of clinical importance. The occurrence of host mutations in KS [26, 27] and the existence of variable levels of KSHV-positive cells might be an indication for the existence of KS spindle cells supported by paracrine mechanisms and/or host oncogenic alternations irreversibly-induced by KSHV. Our results highlight the biological significance of some of these host mutations such as PDGFRAD842V, a mutation that in GIST is known to confer resistance to Imatinib, and mutations occurring in innate immune-regulating genes that were clonally selected during *in vivo* tumorigenesis pointing to their active role in the KSHV oncogenic process. Thus, KSHV-induced host mutations within AIDS-KS lesions could be selected during chemotherapy or targeted therapies and affect their clinical outcomes [3, 51–54].

In summary, our results have uncovered specific aspects of the interplay between host oncogenic alterations and virus-induced transcriptional effects, as well as the epigenetic reprogramming induced by KSHV infection and tumorigenesis. The existence of virally-induced irreversible genetic and epigenetic oncogenic alterations underscores the transforming potential of KSHV infection and supports the possibility of “hit and run” KSHV-sarcomagenesis, which is consistent with findings of LANA-negative spindle-cells in KS lesions. Our results further highlight the potential for the existence of KSHV-induced host mutations that may contribute to the oncogenic process and could be selected upon treatment impacting AIDS-KS therapies.

## Methods

### Cell culture and reagents

mECK36, KSHV (+), cells employed in the present study were originated from frozen batches of mECK36 cells previously generated [21]. KSHV (+) tumors were obtain as previously shown [21], 1x10^6^ KSHV (+) cells were injected subcutaneously into the flanks of nude mice and KSHV (+) tumors formed 5 weeks after injection. KSHV (-) cells were used from frozen populations of KSHV null mECK36 previously obtained after 4 weeks of culturing mECK36 cells without Hygromycin and further selected by weeding and cell sorting [21]. KSHV (-) tumor cells were obtained from frozen stocks previously generated by explanted mECK36 tumor cells that have lost the Bac36-KSHV episome after 4 weeks of culture without Hygromycin [21]. These KSHV-negative cells were further selected by weeding and cell sorting, and characterized thoroughly for KSHV negativity by PCR for LANA, K1, vIRF-1, ORF23, ORF 36, ORF 74, and K15 [23]. KSHV (-) tumors were obtain as previously shown [23], 1x10^6^ KSHV (-) tumor cells were injected subcutaneously into the flanks of nude mice and KSHV (-) formed tumors 3 weeks after injection.

### RNA-Sequencing analysis

RNA was isolated and purified using the RNeasy mini kit (Qiagen). RNA concentration and integrity were measured on an Agilent 2100 Bioanalyzer (Agilent Technologies). Only RNA samples with RNA integrity values (RIN) over 8.0 were considered for subsequent analysis. mRNA from cell lines and tumor samples were processed for directional mRNA-sequencing library construction using the Preparation Kit according to the manufacturer's protocol. We performed paired-end sequencing using an Illumina NextSeq500 platform, all samples were processed in the same sequencing run of Illumina NextSeq 500 system and analyzed together with the aim to avoid the batches effect. The short sequenced reads were mapped to the mouse reference genome (GRCm38.82) by the splice junction aligner TopHat V2.1.0. We employed several R/Bioconductor packages to accurately calculate the gene expression abundance at the whole-genome level using the aligned records (BAM files) and to identify differentially expressed genes between cell lines and cell lines and tumors. Briefly, the number of reads mapped to each gene based on the TxDb. Mmusculus gene ensembls were counted, reported and annotated using the Rsamtools, GenomicFeatures, GenomicAlignments packages. To identify differentially expressed genes between cell lines and tumor samples, we utilized the DESeq2 test based on the normalized number of counts mapped to each gene. Functional enrichment analyses were performed using the ClueGo Cytoscape's plug-in (http://www.cytoscape.org/) and the InnateDB resource (http://www.innatedb.com/) based on the list of deregulated transcripts. Data integration and visualization of differentially expressed transcripts were done with R/Bioconductor. KSHV transcriptome was analyzed using previous resources and KSHV 2.0 reference genome [55], while edgeR test was employed for differential gene expression analysis of KSHV transcripts.

### Methylated DNA Binding Protein sequencing (MBD-sequencing)

#### Methylated DNA enrichment

Genomic DNA was isolated using DNeasy Blood & Tissue Kit (QIAGEN) and sheared by sonication to fragments of ~500bp. The sheared DNA (1μg) was added to 10 μl MBD-Bead slurry (MethylMiner DNA Enrichment Kit, Invitrogen, Carlsbad, CA) and incubated on a rotating mixer for 1 hr., as described previously [56]. The DNA fragments were eluted into distinct subpopulations based on the degree of methylation; non-captured fraction (NC, representing un-methylated DNA fragments), 450 mM NaCl (representing partially methylated DNA fragments) and 2000 mM NaCl (representing methylated DNA fragments). The fractions were then ethanol precipitated and re-suspended in H_2_O.

#### DNA sequencing and analysis

MBD2 enriched methylated DNA fractions were subjected to library preparation with NEBNext Ultra II DNA library prep kit for Illumina (NEB #E7645) at the next generation genomic center in the Azrieli Faculty of Medicine, Bar-Ilan University. The DNA libraries were sequenced on Illumina HiSeq2000, with HiSeq rapid 100PE. Data analysis was performed on the Partek flow platform, raw reads were aligned to the mouse genome mm10 (GRCm38/mm10) with BWA and more than 80% reads were uniquely aligned to the reference genome [57]. Mapped reads were analyzed with MACS2 to generate peaks and annotate differentially methylated regions. Peaks with False discovery rate (FDR) ≤5% & Fold change (FC) ≥2, were considered differentially methylated. When peaks were located between 1500 bp upstream and 200 bp downstream of the transcription start site (TSS), they were considered as differentially methylated gene promoters.

### Immunofluorescence staining

Immunofluorescence assay (IFA) was performed as previously described [21]. Cells were fixed in 4% paraformaldehyde for 10 min and washed with PBS. Cells were permeabilized in 0.2% Triton-X/PBS for 20 min at 4°C. After blocking with 3% of BSA in PBS and 0.1% Tween 20 for 60 min, samples were incubated with Primary antibodies overnight at 4C. After PBS washing, samples were incubated with fluorescent secondary antibodies for 1 hour (Molecular Probes), washed and mounted with ProLong Gold antifade reagent with DAPI (Molecular Probes). Images were taken using a Zeiss ApoTome Axiovert 200M microscope.

### Animal studies

All mice were housed under pathogen-free conditions. Tumor studies were done in 4- to 6- week-old nude mice obtained from the National Cancer Institute. Tumors were generated by subcutaneous injection of mECK36 cells (3 x 10^5^ cells) as previously described [21].

### Clinical tissue analysis

Skin KS biopsies were analyzed from an ACSR (The AIDS and Cancer Specimen Resource) tissue microarray. Immunohistochemistry of clinical tissue microarrays was performed using a standard protocol of the Immunohistochemistry Laboratory of the Department of Pathology at the University of Miami. Antibody staining of p-PDGFRA from R&D Systems (Minneapolis, MN) was diluted to 1:30 and LANA from Abcam (Cambridge, MA) was diluted 1:40.

### Ethics statement

All animal experiments were conducted following NIH guide for the Care and Use of Laboratory Animals. The animal experiments have been performed under UM IACUC approval number 16–093. The University of Miami has an Animal Welfare Assurance on file with the Office of Laboratory Animal Welfare (OLAW), National Institutes of Health. Additionally, UM is registered with USDA APHIS. The Council on Accreditation of the Association for Assessment and Accreditation of Laboratory Animal Care (AAALAC International) has continued the University of Miami’s full accreditation.

## Supporting information

S1 Fig Kaplan-Meier percent tumor-free survival curve from subcutaneous injection of KSHV (-) tumor cells (N = 3) and KSHV (+) tumors (N = 6). (TIF)

S2 Fig KSHV mapped paired reads per million sequenced reads in KSHV (+) cells, KSHV (+) tumors, KSHV (-) cells and KSHV (-) tumors. (TIF)

S3 Fig Immunofluorescence analysis of KSHV LANA (red) in KSHV (+) tumor, nuclei were counterstained with DAPI (blue).
(TIF)

S4 Fig Validation of CpG DNA methylation. Enrichment for methylated DNA was performed on MBD2-beads and the proportion of methylaed DNA was determined by qPCR of the bound versus un-bound fractions for the transition from KSHV(+) cell to KSHV(+) tumor (A) or from KSHV(+) tumor to KSHV(-) tumor (B). Three representative gene promoters were chosen for each transition. Each graph presents results of three biological replicates, n.d (not detected, all DNA came in the un-methylated fraction). Graphs are presented as means + standard deviation, one tailed t tests were performed (*, P ≤ 0.05; **, P ≤ 0.01; ***; P ≤ 0.001).
(TIF)

S1 Table RNA-sequencing and pathways analysis data for gene expression. Tab-A: Differential expressed genes (DEGs) between KSHV (+) and KSHV (-) cells. Tab-FEA1: pathway analysis of DEGs in Tab-A. Tab-B: Differential expressed genes (DEGs) between KSHV (+) tumors and KSHV (+) cells. Tab-FEA2: pathway analysis of DEGs in Tab-B. Tab-C: Differential expressed genes (DEGs) between KSHV (-) tumor cells and KSHV (-) tumors. Tab-FEA3: pathway analysis of DEGs in Tab-C. Tab-D: Differential expressed genes (DEGs) between KSHV (-) tumors and KSHV (+) tumors. Tab-FEA4: pathway analysis of DEGs in Tab-D.
(XLSX)

S2 Table KSHV (+) cell to KSHV (+) tumor Hypo-methylated. (XLSX)

S3 Table KSHV (+) cell to KSHV (+) tumor Hyper-methylated. (XLSX)

S4 Table Biological processes and pathways identified in GREAT during the transition from KSHV (+) cells to KSHV (+) tumors. (TIF)

S5 Table Methylation and expression analysis data. (XLSX)

S6 Table KSHV (+) tumor to KSHV (-) tumor Hyper-methylated. (XLSX)

S7 Table KSHV (+) tumor to KSHV (-) tumor Hypo-methylated. (XLSX)

S8 Table Biological processes and pathways identified in GREAT during the transition from KSHV (+) tumors to KSHV (-) tumors. (TIF)

S9 Table Mutational profiles for all samples included in the current study. (XLSX)
