# Supplementary figures and images for: High-throughput sequencing analysis of a “hit and run” cell and animal model of KSHV tumorigenesis
Source: PLoS Pathog. 2020 Jun 30;16(6):e1008589. doi: 10.1371/journal.ppat.1008589 (PMC7357787; doi:10.1371/journal.ppat.1008589)

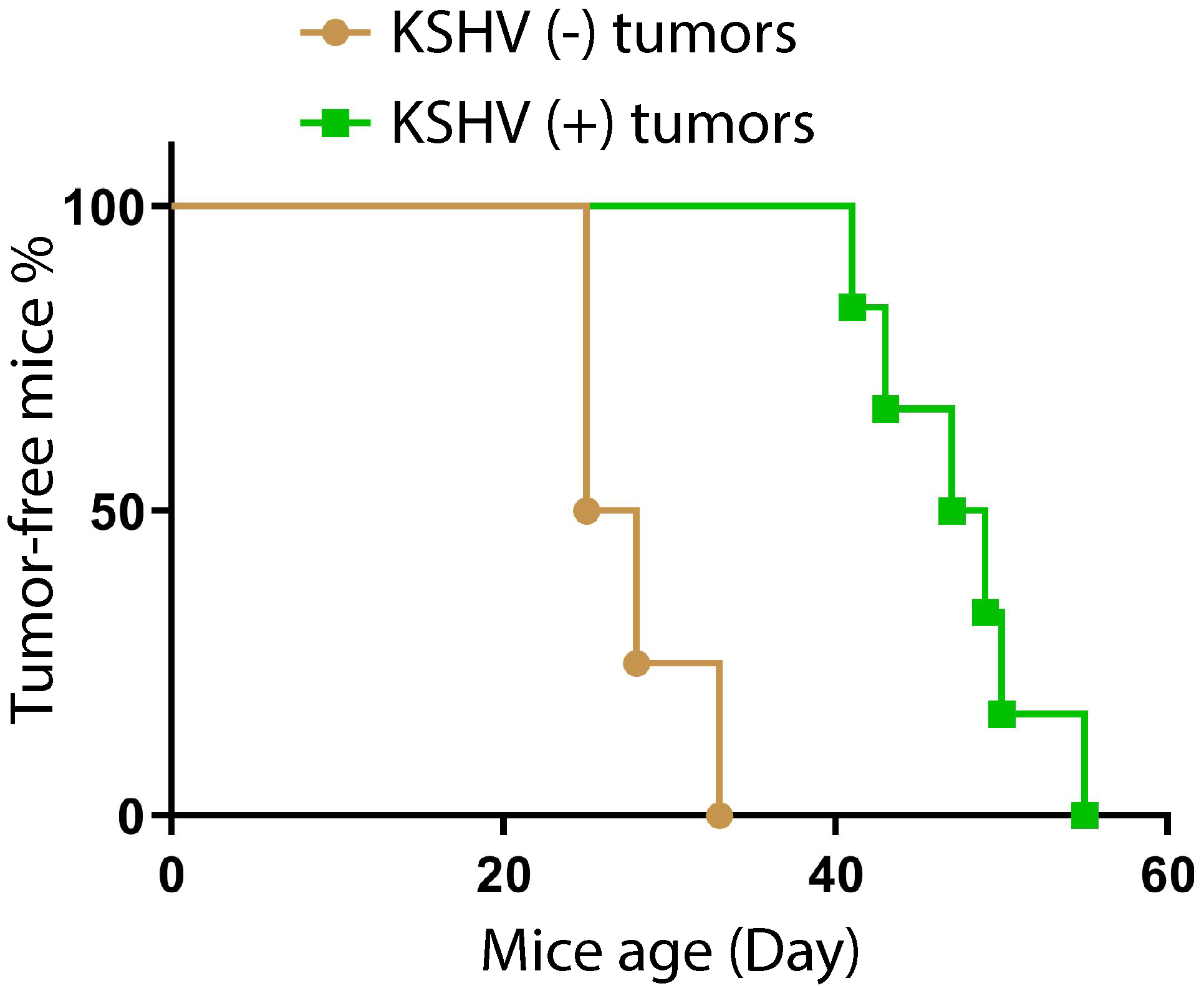

Supplement: S1 Fig — (TIF) [file ppat.1008589.s001.tif]

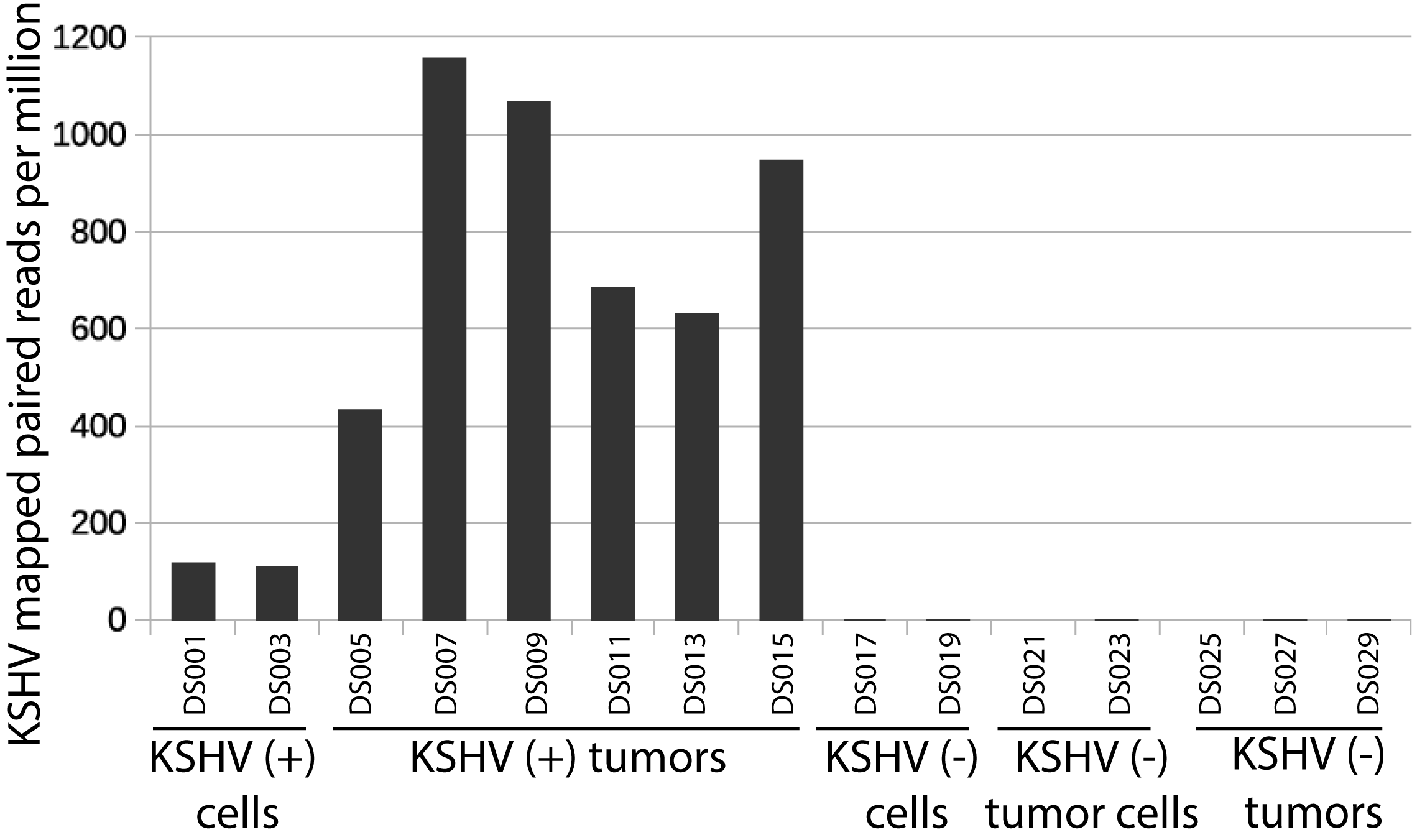

Supplement: S2 Fig — (TIF) [file ppat.1008589.s002.tif]

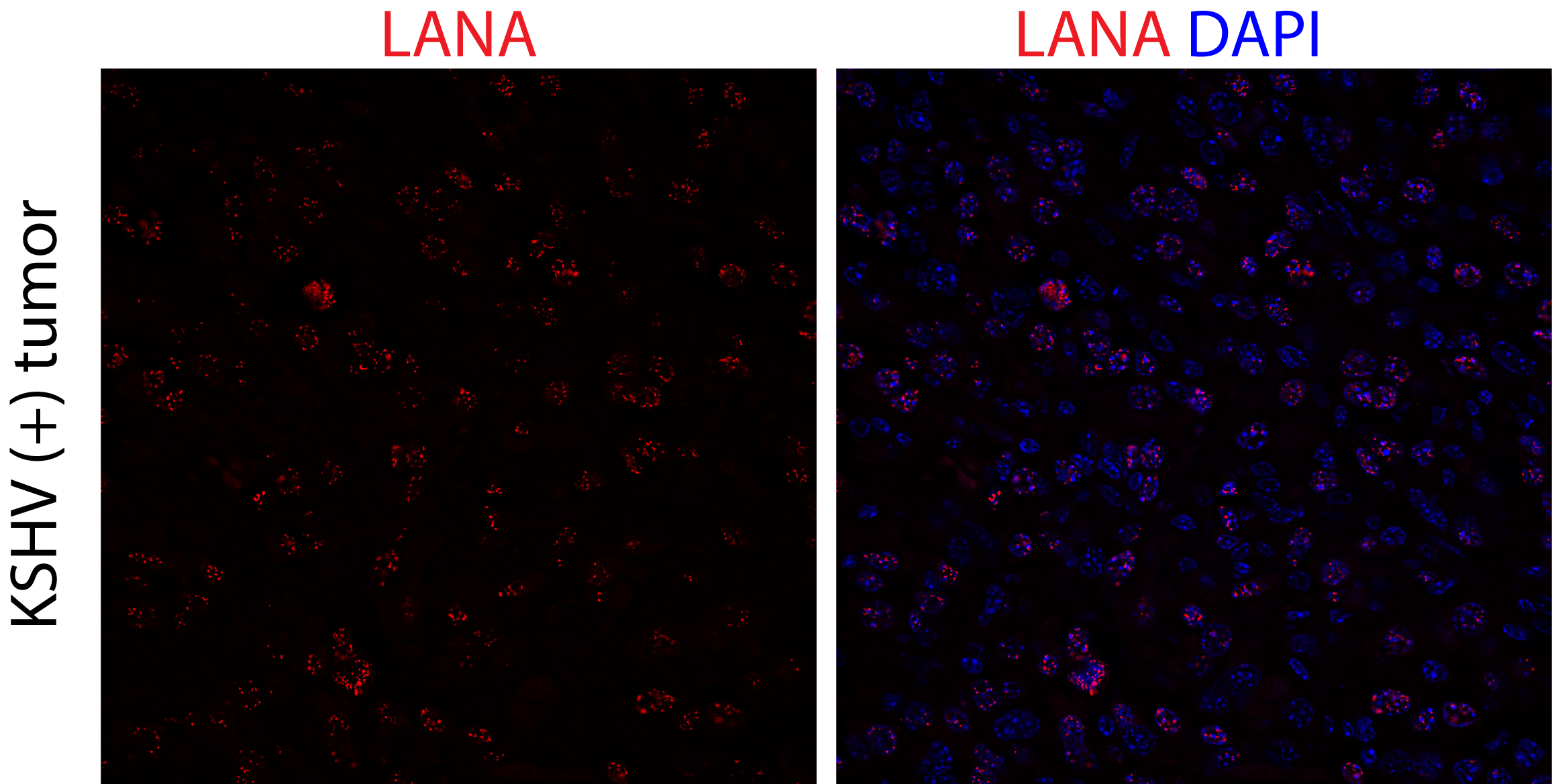

Supplement: S3 Fig — Immunofluorescence analysis of KSHV LANA (red) in KSHV (+) tumor, nuclei were counterstained with DAPI (blue). (TIF) [file ppat.1008589.s003.tif]

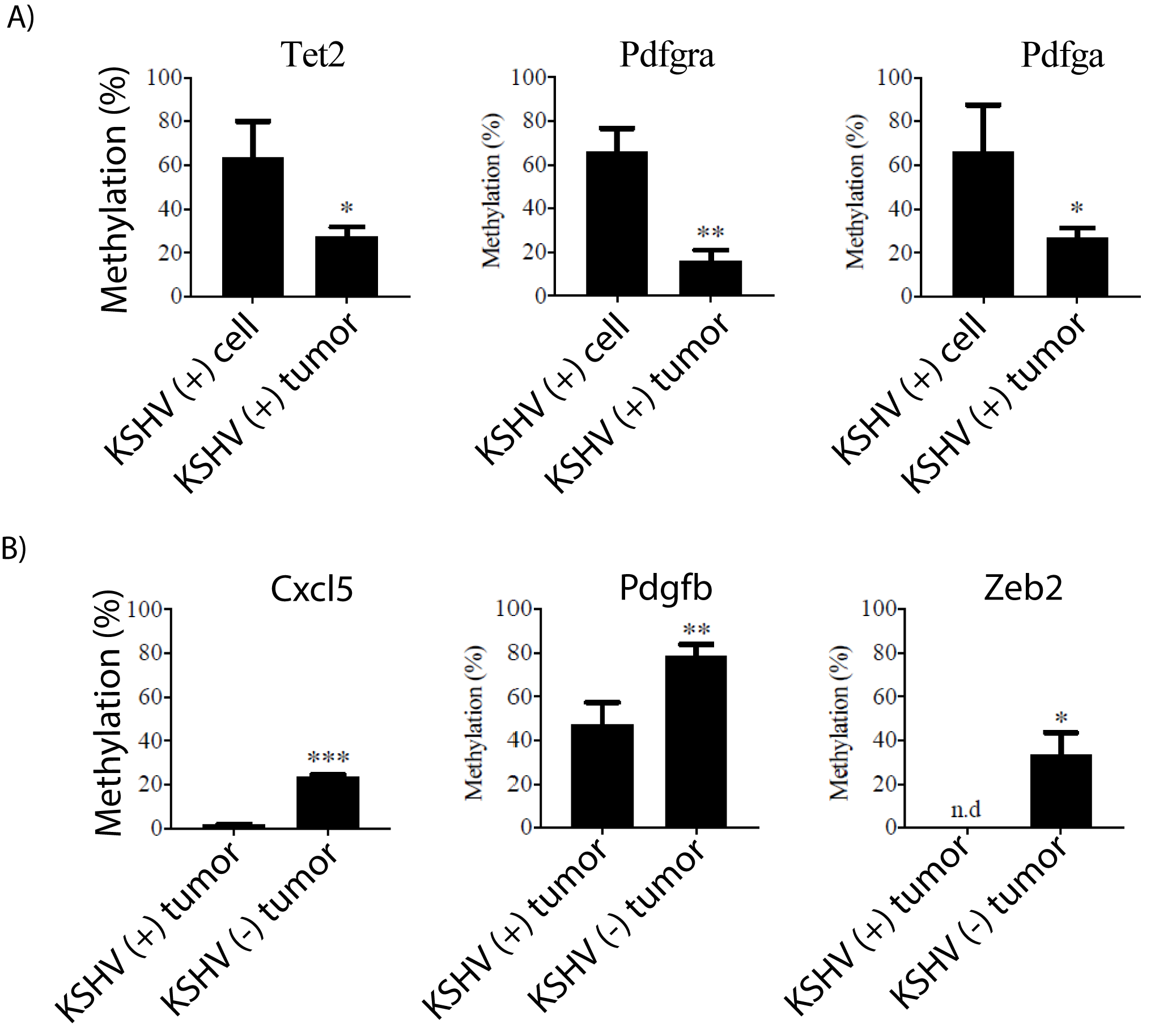

Supplement: S4 Fig — Enrichment for methylated DNA was performed on MBD2-beads and the proportion of methylaed DNA was determined by qPCR of the bound versus un-bound fractions for the transition from KSHV(+) cell to KSHV(+) tumor (A) or from KSHV(+) tumor to KSHV(-) tumor (B). Three representative gene promoters were chosen for each transition. Each graph presents results of three biological replicates, n.d (not detected, all DNA came in the un-methylated fraction). Graphs are presented as means + standard deviation, one tailed t tests were performed (*, P ≤ 0.05; **, P ≤ 0.01; ***; P ≤ 0.001). (TIF) [file ppat.1008589.s004.tif]

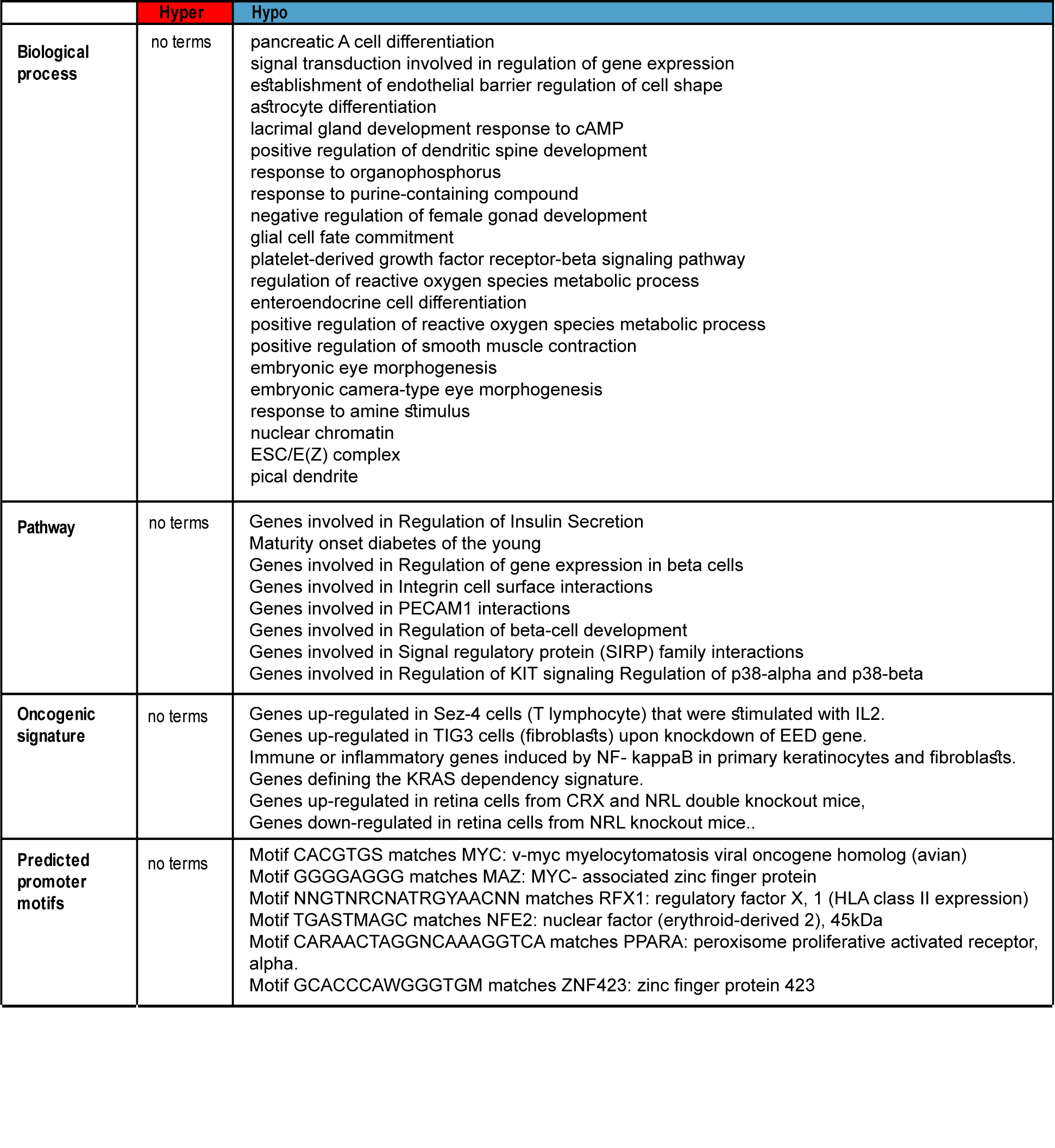

Supplement: S4 Table — (TIF) [file ppat.1008589.s008.tif]

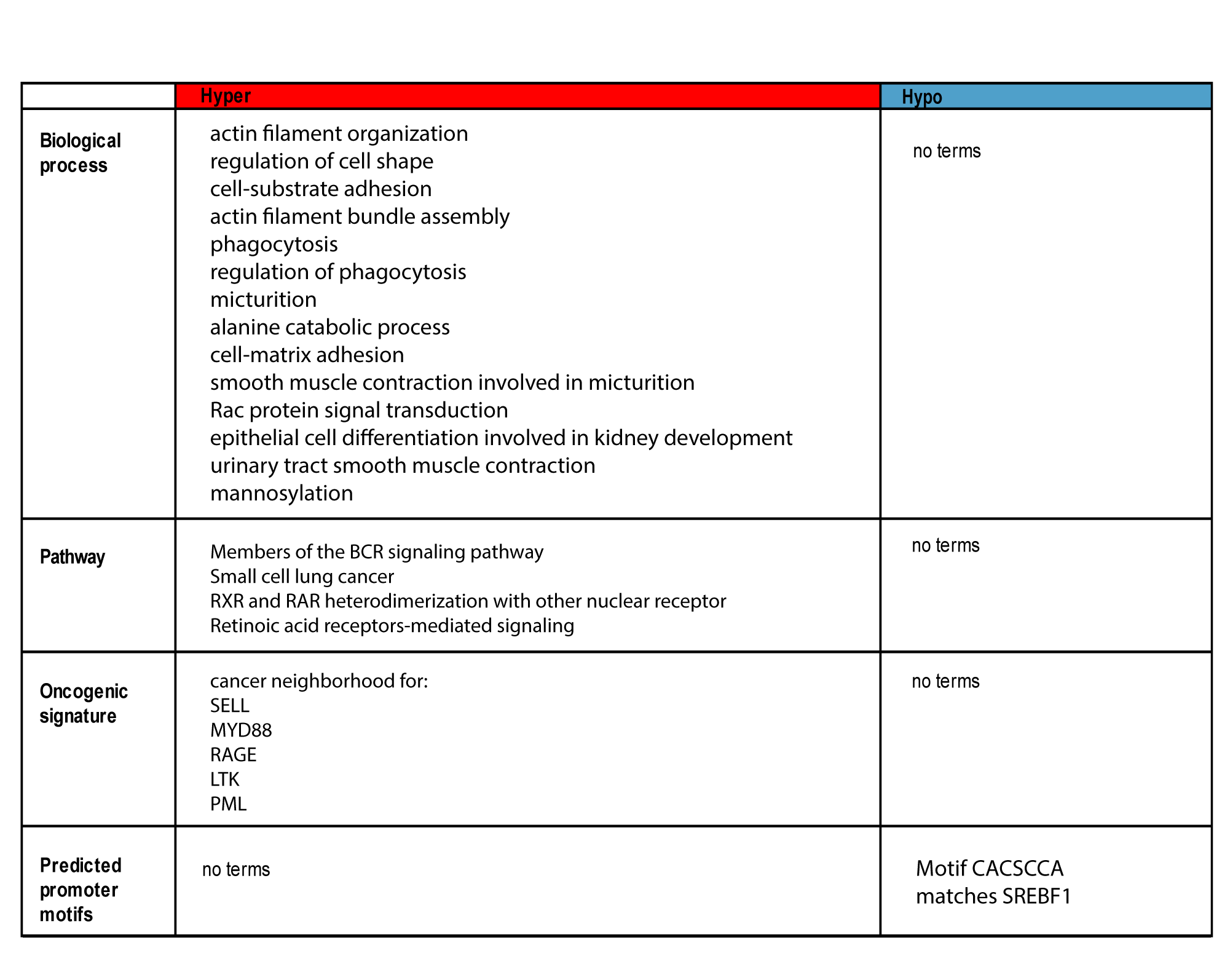

Supplement: S8 Table — (TIF) [file ppat.1008589.s012.tif]
